# Evaluating the potential of residual Pap test fluid as a resource for the metaproteomic analysis of the cervical-vaginal microbiome

**DOI:** 10.1038/s41598-018-29092-4

**Published:** 2018-07-18

**Authors:** Somaieh Afiuni-Zadeh, Kristin L. M. Boylan, Pratik D. Jagtap, Timothy J. Griffin, Joel D. Rudney, Marnie L. Peterson, Amy P. N. Skubitz

**Affiliations:** 1 Department of Laboratory Medicine and Pathology, University of Minnesota, Minneapolis, MN USA; 2 Department of Biochemistry, Molecular Biology and Biophysics, University of Minnesota, Minneapolis, MN USA; 3 Center for Mass Spectrometry and Proteomics, University of Minnesota, Minneapolis, MN USA; 4 Department of Diagnostic and Biological Sciences, School of Dentistry, University of Minnesota, Minneapolis, MN USA; 5 School of Pharmacy, University of Wyoming, Laramie, WY USA

## Abstract

The human cervical-vaginal area contains proteins derived from microorganisms that may prevent or predispose women to gynecological conditions. The liquid Pap test fixative is an unexplored resource for analysis of microbial communities and the microbe-host interaction. Previously, we showed that the residual cell-free fixative from discarded Pap tests of healthy women could be used for mass spectrometry (MS) based proteomic identification of cervical-vaginal proteins. In this study, we reprocessed these MS raw data files for metaproteomic analysis to characterize the microbial community composition and function of microbial proteins in the cervical-vaginal region. This was accomplished by developing a customized protein sequence database encompassing microbes likely present in the vagina. High-mass accuracy data were searched against the protein FASTA database using a two-step search method within the Galaxy for proteomics platform. Data was analyzed by MEGAN6 (MetaGenomeAnalyzer) for phylogenetic and functional characterization. We identified over 300 unique peptides from a variety of bacterial phyla and *Candida*. Peptides corresponding to proteins involved in carbohydrate metabolism, oxidation-reduction, and transport were identified. By identifying microbial peptides in Pap test supernatants it may be possible to acquire a functional signature of these microbes, as well as detect specific proteins associated with cervical health and disease.

## Introduction

The proteome of cervical-vaginal fluid (CVF) is extremely complex, containing proteins predominantly synthesized by the endocervix and vaginal cells, but also from the endometrial and tubal secretions and from the peritoneal fluid^1^. In addition to host proteins, the CVF also contains diverse microbial communities resulting from microbes that colonize the cervical-vaginal area^2–4^. The composition of the human vaginal microbiome varies between women, and under different conditions, such as in pregnancy and after menopause, and may contribute to preterm birth and genitourinary symptoms of menopause^5–8^. The dominance of *Lactobacilli* or other lactic acid producing bacteria may protect against infection with HIV and other sexually transmitted diseases^9^. However, the role of the cervical-vaginal microbiota in the etiology of most gynecological conditions is not well understood^10,11^.

New molecular techniques have made it possible to study microbes without growing them in culture^12–14^. The studies of the NIH Human Microbiome Project (HMP)^15–17^ have vastly increased the diversity of the bacteria identified in the human vagina over culture-dependent methods, and the development of a comprehensive database of 16 S rRNA sequences from common vaginal bacteria has allowed species level classification of bacteria from clinical samples^18^. These molecular approaches yield detailed information on microbiome composition and the abundance of bacterial taxa present, however the functional significance of diverse microbial communities and their interaction with host proteins in differing states of health and disease is lacking.

Metaproteomic analysis provides a complementary approach to DNA-based phylogentic profiling, yielding information on the structure of polymicrobial communities, as well as protein expression and function. Using mass spectrometry based proteomic techniques, in combination with predicted protein databases based on high throughput sequencing^17^, has the potential to yield important information on host-microbial interactions in healthy and diseased states. In particular, the metaproteome of extracellular microbial proteins may be important for understanding host-microbiome interactions and response to environmental stimuli^19,20^.

We previously demonstrated the feasibility of using the residual fixative from discarded Pap tests for the identification of human cervical-vaginal proteins by mass spectrometry^21^. Screening for cervical cancer by Pap tests has been performed routinely for over 50 years^22^ and over 30 million are performed annually in the U.S^23^. The liquid-based Pap test consists of collecting cervical cells from the external cervical os and placing them into a vial containing an alcohol-based fixative^24,25^. After processing the cells from the vials and staining them, they are examined by a pathologist to identify the presence of premalignant and malignant cells. Liquid-based Pap tests may also be used for the identification of human papilloma virus infection^26,27^ and the genomic amplification of the human telomerase gene, both of which are associated with the development of cervical cancer^27–29^. The liquid fixative solution in which the cells are collected for Pap tests is discarded after examination of the cells. Using this unique sample source, we identified over 400 proteins from the Human Uniprot database, and defined a “Normal Pap test Core Proteome” consisting of 153 proteins^21^. To evaluate this approach for the identification of taxonomic and functional information on the vaginal microbiome, these same raw data were analyzed using a two-step method for peptide sequence matching and protein identification for metaproteomic analysis of microbial peptides present in the residual Pap test fixative from women over age 50.

## Results

**Figure 1:**
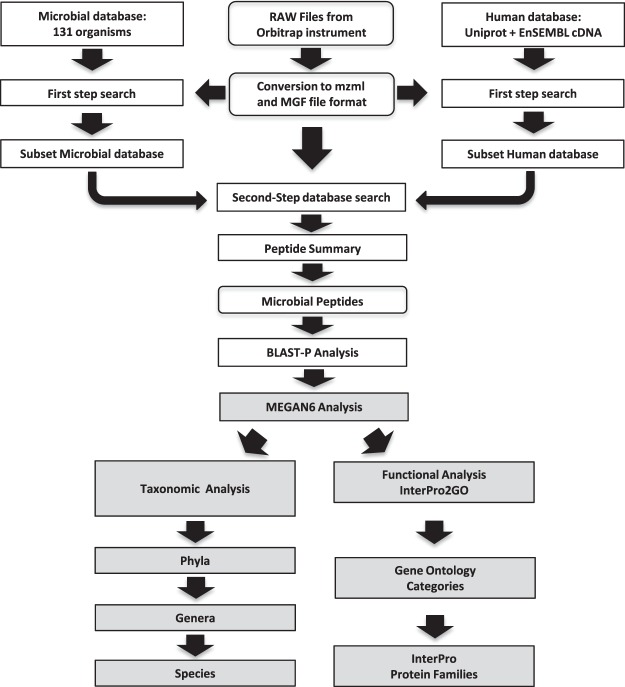
Flow chart for the two-step database search using Galaxy-P. The first step database search uses low-stringency scoring to create a smaller, refined database. In the second step, files were searched using a “target-decoy” version of the refined database at high stringency. A list of microbial peptides was filtered from the Peptide summary of the second step search and used to search with BLASTP. The results of BLASTP searches of high confidence microbial peptides were used as input for the metagenomic/metaproteomic analyzer MEGAN6 for taxonomic and functional characterization. See methods for details.

High accuracy MS/MS data from residual Pap test supernatants was searched using a two-step method^30,31^ against a database composed of microbial and human protein sequences. The database of microbial species of the urogenital tract [130 bacterial proteomes plus *Candida* (Supplementary Table S1)] was generated from Human Microbiome Project reference genomes^32,33^. The database search was performed with ProteinPilot™ within the Galaxy for proteomics (Galaxy-P) platform using the workflow depicted in Fig. 1. High confidence microbial peptide matches were analyzed by using the downstream metagenomic/metaproteomic analysis tool MEGAN6^34^ for taxonomic assignment and functional characterization.

### Phylogenetic analysis of pap test samples

Microbial peptides identified in the residual Pap test fluid from five individual samples, and a pool of samples from 40 women, were analyzed using MEGAN6 to assign peptides to microbial taxa. Of the 1463 total peptide spectral matches (PSMs) analyzed, 50% were assigned to phyla, 42% to genera and 15% to species (Table 1).

**Table 1:** Taxonomic assignments by MEGAN6.

	Number of Peptide Spectral Matches assigned by MEGAN6*						
	Npap94	Npap134	Npap137	Npap929	Npap933	Npap Pool	Total
BLASTP (Input)^†^	36	53	64	149	875	286	1463
Phylum	21	24	34	67	434	156	736
Genus	19 (16)	20 (17)	28 (18)	55 (28)	353 (200)	135 (88)	610 (367)
Species	10	15	10	39	77	67	218
InterPro Families	13	18	22	86	755	165	1059

*Numbers in parenthesis correspond to the number of unique peptide assignments.

^†^Indicates the number of “high confidence” microbial peptides submitted to BLAST. Taxonomic assignments made by MEGAN6 when parsing BLAST results may exclude some of those peptides, depending on how the LCA parameters are set.

In the pooled sample, we found 156 PSMs belonging to 6 bacterial phyla (*Actinobacteria*, *Bacteroidetes*, *Firmicutes*, *Fusobacteria*, *Proteobacteria*, and *Spirochaetes*) and the phylum *Ascomycota* (fungi) (Table 2). The number of PSMs assigned to phyla in four of the five individuals ranged from 21 to 67, while in sample NPap933 we found 434 PSM assignments. Of the seven bacterial phyla identified, *Firmicutes*, *Actinobacteria*, and *Proteobacteria*, were identified by at least 2 PSMs in all samples (Table 2), with *Firmicutes* representing the largest phylum. *Bacteroidetes* was present at moderate levels, with at least 2 PSMs in the pooled sample and three of the individual samples. The remaining (minor) phyla were represented by a single PSM in the pooled sample, with 1–5 assignments in two or three of the individual samples.

**Table 2:** Numbers of reads assigned at the Phylum level by MEGAN6.

Phylum	Peptide Spectral Matches assigned to Phyla					
	Npap94	Npap134	Npap137	Npap929	Npap933	NPapPool
Firmicutes	8	15	15	14	388	110
Actinobacteria	9	4	8	35	26	32
Proteobacteria	4	2	4	9	8	8
Bacteroidetes			2	4	10	3
Spirochaetes				5	1	1
Fusobacteria		1	4			1
Ascomycota		2	1		1	1
TOTAL	21	24	34	67	434	156

**Figure 2:**
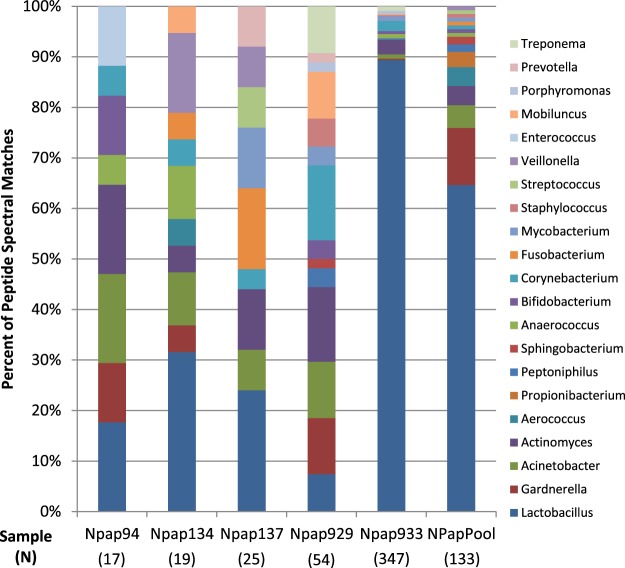
Comparison of the 21 genus level assignments for individual and pooled Pap test samples. Five individual Pap samples and the NPap pool were compared by genus level assignments based on the number of PSMs for genera with two or more PSMs. NPap933 and the NPap pool and were dominated by matches to *Lactobacillus*; the four other individual samples were more diverse. N represents the total number of PSMs per sample assigned to genera with at least two PSMs. See Supplementary Table S2 for a complete listing of PSM assignments to genera.

Using a liberal threshold (1 PSM) we identified 30 genera in the pooled and individual Pap test samples (Supplementary Table S2). When we used a moderate threshold of 2 PSMs in at least one sample, the number of genera with PSMs was reduced to 21 (Fig. 2). By applying an even more stringent filter of 2 peptides in one or more samples, the number of genera identified was 18 (five in the pooled sample and 16 in the combined individual samples) (Supplementary Table S2). *Lactobacillus* was the only genus to meet the 2 peptide threshold in all six samples, and was also the genus with the most peptides assigned in all samples except NPap929. In the pooled sample, *Gardnerella* was the second most abundant genus (10 peptides), however *Gardnerella* did not meet the threshold in any of the individual samples. In the individual samples, the second most abundant genera were *Corynebacterium*, *Acinetobacter*, and *Actinomyces*. Of these, *Actinomyces* did not meet the two peptide threshold in the pooled sample.

**Figure 3:**
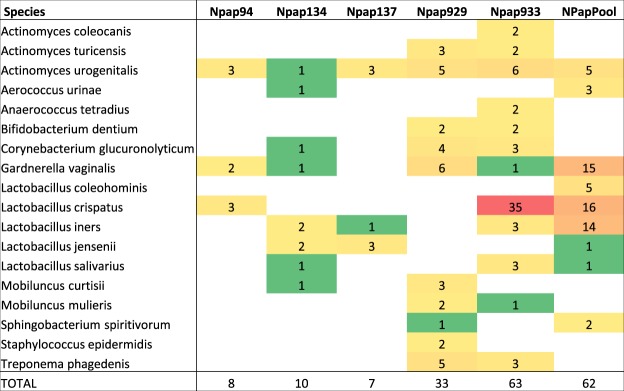
Heat map of species level assignments for individual Pap samples and NPap pool. Shown are the PSM assignments to bacterial species with 2 or more PSM assignments in at least one sample. Green = 1; Yellow = 2–10; Orange = 11–30; Red = >30. See Supplementary Table S4 for a complete listing of PSM assignments to species.

At the species level, 218 of the 1463 input PSMs (15%, Table 1) were assigned to 43 bacterial species and the yeast *Candida albicans*. Using a moderate threshold, 183 assignments were made to 18 bacterial species; 7 were present in the pooled sample and 15 species in one or more of the individual samples (Fig. 3; Supplementary Table S3). Overall, five *Lactobacillus* species were identified, three *Actinomyces* species, and two *Mobiluncus* species. Only two species level assignments were made to NPap137, while NPap933 had 10 species level assignments, more than any individual or the pooled sample. Three species were unique to the NPap pool, having only one or no PSM assignments in the individual Pap test samples, while eleven species were identified in one or more of the individual samples, but not in the pooled sample. No *Lactobacillus* species were identified in the individual sample NPap929, unlike the other individuals. In contrast, this individual also had a higher number of PSM assignments to *Gardnerella vaginalis*. *Actinomyces urogenitalis* was the only species with at least two assignments in four of the five of the individual samples as well as the pooled sample.

### Functional analysis of the metaproteome from pap test samples

MEGAN6 was used on BLASTP searches of protein sequences to generate functional characterization of the metaproteome and to assign peptides to protein families and Gene Ontology categories using InterPro2GO. Overall, 113 protein families in 54 Gene Ontology categories were identified with at least two PSMs (Supplementary Table S4 and Supplementary Table S5). The Gene Ontology categories for biological processes are shown in Table 3. In the pooled Pap test sample, 143 PSMs were mapped to 32 protein families in 29 Gene Ontology categories (Supplementary Table S4 and Supplementary Table S5). Seven of the top 20 protein families with the most PSM assignments were associated with glycolysis and lactic acid production, suggesting that the organisms identified were metabolically active^35^. In total, nine of the 32 protein families identified in the pooled sample were associated with glycolysis. Other major protein families included: ribosomal proteins and proteins involved in transcription or translation, and proteins associated with DNA metabolism. The Gene Ontology categories for biological processes with the most PSM assignments were for oxidation-reduction processes, carbohydrate metabolism, and small molecule metabolism (Table 3).

**Table 3:** List of the Gene Ontology Categories for Biological Process with two or more Peptide Spectral Matches*.

GO Category	Biological Process
**GO:0046034**	**ATP metabolic process**
**GO:0009058**	**biosynthetic process**
**GO:0005975**	**carbohydrate metabolic process**
**GO:0009056**	**catabolic process**
**GO:0006520**	**cellular amino acid metabolic process**
**GO:0016043**	**cellular component organization**
GO:0006935	chemotaxis
**GO:0051186**	**cofactor metabolic process**
**GO:0006259**	**DNA metabolic process**
**GO:0006091**	**generation of precursor metabolites and energy**
GO:0006811	ion transport
**GO:0006807**	**nitrogen compound metabolic process**
GO:0071941	nitrogen cycle metabolic process
**GO:0009117**	**nucleotide metabolic process**
**GO:0055114**	**oxidation-reduction process**
**GO:0016310**	**phosphorylation**
GO:0019538	protein metabolic process
**GO:0006950**	**response to stress**
GO:0016070	RNA metabolic process
GO:0007165	signal transduction
**GO:0044281**	**small molecule metabolic process**
GO:0006790	sulfur compound metabolic process
**GO:0006412**	**translation**
**GO:0006810**	**transport**

*Complete list of Gene Ontology Categories is in Supplemental Table S5.

Bold text indicates Gene Ontology Categories identified in the NPap pool.

The individual Pap test samples were also analyzed using the InterPro2GO database to assign peptides to protein families and Gene Ontology categories. Eleven protein families had at least two PSM assignments in two or more individual NPap samples (Supplementary Table S5). Unlike the pooled Pap test sample, no proteins associated with glycolysis were identified in multiple individual samples, although nine glycolysis proteins had assignments from NPap933 (Supplementary Table S5). In addition, over 40 ribosomal protein families were identified exclusively in NPap933. Interestingly, FliO, a protein involved in flagellar biosynthesis, was identified in several of the individual samples, but not in the pooled sample or in NPap933. Similar to the pooled Pap test sample, the Gene Ontology categories with the most assignments in multiple individual samples were oxidation-reduction and carbohydrate metabolism (Table 3). The categories for biosynthetic process, RNA and cellular amino acid metabolic processes were more prevalent in the individual Pap test samples than in the pool.

### Peptides identified in multiple pap test samples

Our metaproteomic analysis of five individuals and one pool of samples from 40 women yielded a total of 367 microbial peptides assigned to 30 genera (Supplementary Table S2). Twenty-two peptides were identified in more than one sample. Table 4 shows the peptide sequences and the proteins to which they were matched, as well as the nine genus assignments. Twelve out of the twenty-two peptides were from *Lactobacillus*, including two unique peptides from phosphoglycerate kinase and three unique peptides from glyceraldehyde-3-phosphate dehydrogenase. Two structural proteins from *Lactobacillus*, 50 S ribosomal protein L7/L12 and S-layer protein, were also identified in two samples. Haloacid dehalogenase peptides from *Gardnerella* and *Viellonella* were also identified in two samples.

**Table 4:** Peptides identified in multiple samples.

Peptide	Protein	Genus	Species
AEDADDLSPSIVVSR	ABC transporter permease	Actinomyces	A. urogenitalis
SLMLGKEGEGLK	acyl-CoA dehydrogenase	Acinetobacter	A. baumanii, A. species
QAAAAPTQPAPK	sel1 repeat family protein	Acinetobacter	A. baumanii
MLLFAGGDLR	hypothetical protein HMPREF9241_00652	Actinomyces	A. turicensis
DGIEPILEK	ABC superfamily ATP-binding cassette transporter -binding protein	Corynebacterium	C. glucuronolyticum
LEEAGLTKNK	transcriptional regulator	Fusobacterium	F. species
SAGLNPVKSCK	HAD family hydrolase, haloacid dehalogenase	Gardnerella	G. vaginalis
SEFDVELTEAGQEK	50 S ribosomal protein L7/L12	Lactobacillus	L. crispatus, L. species
EADYIVPTTAELK	beta-phosphoglucomutase	Lactobacillus	L. crispatus
LAFFPDDVDHD	enolase	Lactobacillus	Lactobacillus species
VPVPDGSETELVSILSK	glyceraldehyde-3-phosphate dehydrogenase (type I)	Lactobacillus	Lactobacillus species
YDTTHGTFNHEVSSTEDSIVVDGK	glyceraldehyde-3-phosphate dehydrogenase (type I)	Lactobacillus	Lactobacillus species
TIHAYTGTQMTLDGPSR	glyceraldehyde-3-phosphate dehydrogenase (type II)	Lactobacillus	Lactobacillus species
DNVAATEFSNDASR	phosphoglycerate kinase	Lactobacillus	L. crispatus
LIVDDLDVK	phosphoglycerate kinase	Lactobacillus	Lactobacillus species
AVVANSAEEANSK	pyruvate kinase	Lactobacillus	L. crispatus, L. species
DGDTVYVADQTR	S-layer protein/surface layer protein	Lactobacillus	L. crispatus, L. species
DLYNEETAENVR	triose-phosphate isomerase	Lactobacillus	L. crispatus, L. species
QAEYDLITK	YjcQ protein	Lactobacillus	L. iners
ITWGEMEK	type VII secretion protein EccC	Mycobacterium	Mycobacterium species
EAAIAFSAIEK	GTP-binding protein YchF/ribosome binding ATPase	Treponema	T. phagedenis
IADLQWVDGAK	HAD family hydrolase, haloacid dehalogenase	Veillonella	V. atypica

## Discussion

This study evaluates the use of residual Pap test samples for taxonomic and functional characterization of bacteria present in the female genital tract using a metaproteomic approach. Numerous studies using approaches such as 16 S rRNA gene sequencing have examined the composition of the vaginal microbiome relative to various states of health and disease^9,11,36–40^, while relatively few have examined the bacterial proteins expressed in the cervical-vaginal area^41–44^.

Our metaproteomic analysis of five individuals and one pool of samples from 40 women yielded 610 PSMs (representing 367 unique microbial peptides) from cell-free Pap test fluid using a two-step search method to increase the number of high confidence matches^30,31,45,46^. Using a two-step database search method has several advantages, it: (a) allows us to use large databases in the first step, (b) generates comprehensive reduced databases with the correct protein composition, and (c) assigns spectra to peptides correctly by having a protein database generated from a tandem/hybrid search method^30^.

The FDR threshold that we used for the second step search was 5% local FDR. Local FDR measurements, which are part of the ProteinPilot output, measure the error rate for individual PSMs, peptides or proteins. This is in contrast to the global FDR measurements which estimate the error rate for the entire set of PSMs, peptides or proteins. We have used local FDR for peptides since it assures that all reported peptide identifications have at least some minimal quality. In general, 5% local FDR threshold is much more stringent than 1% global FDR threshold and also facilitates better comparison across datasets^47^.

With a moderately stringent filter of 2 PSMs, assignments were made to 34 phylotypes, including 21 genera and 18 bacterial species (Supplementary Table S6). This amounted to 23% of the species in our database, which was composed of 131 proteomes representing 77 microbial species of the urogenital tract from the Human Microbiome Project reference genomes. Using a liberal filter of 1 PSM increased the number of species identified to 44. Most of the PSM assignments were made to the genus *Lactobacillus*; five *Lactobacillus* species were assigned at least two PSMs. Three or more peptides from *Lactobacillus* species were identified in each of the samples examined. Three other genera (*Acinetobacter*, *Actinomyces and Corynebacterium*) were also identified in all five of the individual Pap samples, as well as the pooled sample.

At the species level, *Actinomyces urogenitalis* was the only bacterial species with PSM assignments found in all 6 samples. In 16S rRNA gene analyses of the vaginal microbiome in women without symptoms of infection or bacterial vaginosis, *Actinomyces urogenitalis* was detected, but it was not common^3,48^. *Gardnerella vaginalis* peptides were identified in all but one of our samples. The presence of *Gardnerella vaginalis* has previously been associated with bacterial vaginosis^48,49^; however the cytology results from our samples reported no overt signs of infection.

Previous 16S rRNA analyses have examined the species composition of the vaginal bacterial communities present in healthy women and found that they cluster into five groups based on species composition and abundance. These groups have been termed community state types (CST)^3,37,48,50^. Four of these vaginal bacterial communities are dominated by a single species of *Lactobacillus*, while the fifth CST is more diverse, containing lower numbers of *Lactobacilli* and more species of strictly anaerobic bacteria)^3,37,48,50^. Overall, we identified 9 different *Lactobacillus* species with at least 1 PSM assignment, including *L*. *crispatus*, *L*. *iners*, and *L*. *jensenii*, which define CST I, III, and V, respectively. No assignments were made to *L*. *gasseri*, which is the major component of CST II. Many CST IV species, including *Prevotella*, *Dialister*, *Atopobium*, *Gardnerella*, *Aerococcus*, *Anaerococcus*, *Peptoniphilus*, and *Mobiluncus*, were identified in our study. Other CST IV species (*Eggerthella*, *Sneathia* and *Finegoldia*) were not identified; however, of these, only *Finegoldia* sequences were present in our database. Based on genera level assignments, NPap933 is the individual sample that most closely resembles the pool of samples from 40 women, due to the large number of *Lactobacillus* assignments, while the other four individual samples are more diverse. Using the MEGAN6 species level assignments to classify samples into CSTs, we observed that over 30% of the peptides in the NPap pool could be assigned to the low- *Lactobacillus*, high species diversity CST IV. While it is possible that the results from our pooled sample are skewed by the dominance of a single protein-rich sample (such as NPap933), our results are still comparable to what has been observed in 16 S rRNA studies of post-menopausal women, where over 45% of the women were classified to the low-*Lactobacillus* CST IV^50^. Although our studies did not classify women by menopausal status, the median age for samples in the pool was 58 years. For the individual samples, two (NPap134 and NPap929) could potentially be classified as diverse (CST IV) based on the number of species with peptides assigned, while two samples had a higher percentage of peptides assigned to either *L*. *crispatus* (CST I, NPap94) or *L*. *jensenii* (CST V, NPap137). These classifications must be viewed cautiously due to the low number of total assignments in these samples. In contrast, NPap933 had assignments to 26 species and appears diverse compared to the other individual samples; 45% of the PSMs from this sample were assigned to *L*. *crispatus* (CST I). The apparent diversity of NPap933 is likely due to the high number of peptides assigned compared to the other individuals.

Earlier work on the taxonomy of the vaginal microbiota relied on analysis of whole genome sequencing or qPCR using primers in conserved regions^3,18,37,48,50^. While a complete representation of the community structure of the vaginal microbiome using metaproteomics (in particular at the species level) is limited by peptide sequence conservation, several recent studies have used metaproteomics^43^ or a combination of metaproteomics and metagenomics^44^ to classify cervical lavage samples into *Lactobacillus*-dominated and *Gardnerella vaginalis*-dominated community groups. Zevin *et al*.^44^, confirmed their metaproteomic bacterial community composition by 16 S rRNA gene sequencing and found that although the diverse community group was still dominated by *Gardnerella*, more bacterial species diversity and lower levels of *Gardnerella* were found by16S rRNA sequencing than by using mass spectrometry^44^. We attempted a similar comparison to our metaproteomic data using DNA extracted from the stored cell pellet of the individual Pap samples. Unfortunately, the results of PCR amplification and sequencing of 16 S rRNA genes were inconclusive, perhaps due to the low sample biomass. Another recent examination of potential and active functions in the gut microbiome found the metagenomic and metaproteomic taxonomic classifications were similar^51^. However, the most abundant taxa were not necessarily the most metabolically active, and several taxa in particular exhibited a high level of protein expression relative to their abundance^51^. Future studies of the microbiota from Pap test samples will focus on sample preparation methods that would allow both mass spectrometry as well as metagenomic and 16 S rRNA gene analyses.

In our study, we identified 32 microbial protein families in the pooled sample. However, only a single protein from the pool (enolase) was also identified in most of the individual samples. One explanation for this may be the dominance of *Lactobacillus* species present in the pool compared to the individual samples. In their study of the *L*. *iners* and *L*. *crispatus* proteomes derived from cervicovaginal lavage samples, Borgdorff *et al*.^41^ identified many of the same proteins we identified in our sample of pooled residual Pap tests. They identified 40 *Lactobacillus* proteins, seven of which we also found in our residual Pap test samples; including five protein families associated with glycolysis (enolase, fructose-1,6-bisphosphate aldolase, glyceraldehyde phosphate dehydrogenase, phosphoglucose isomerase, and pyruvate kinase) as well as the DNA-binding protein Dps, and the translation elongation factor EFTu. Six of these seven *Lactobacillus* proteins had assignments in NPap933 or the pooled Pap sample; both of which had approximately half of their PSMs assigned to *L*. *crispatus* or *L*. *iners*. In contrast, the flagellar biosynthetic protein, FliO, was identified in the four individual samples with more diverse taxonomy, but not the *Lactobacillus* dominant NPap933 or the pooled Pap sample. FliO is part of the flagellar export apparatus and is required for efficient flagellar biosynthesis in *Salmonella enterica*. Although it is less conserved than other flagellar export proteins, a homolog of FliO exists for many species of *Proteobacteria*^52,53^.

It is not clear why the number of peptides assigned varied so greatly between NPap933 and the samples from the four other individuals. In our previous analysis of human proteins present in residual Pap test fixative, we identified between 317 and 500 proteins from these same Pap samples^21^. While it is tempting to think that this might reflect a difference in the relative abundance of bacterial colonization of those individuals, the low number of microbial peptides recovered from individual samples NPap94, NPap134, and NPap137 could also be due to limitations in the way those samples were collected and processed. Further optimization may be needed to improve sample collection and processing protocols in order to increase the number of PSMs and taxa recognized. Two studies comparing the cervical “cytobrush” (used for Pap tests) to other collection devices for analysis of microbiome composition found the cytobrush to be as good^54^ or better than rayon swabs for collecting higher bacterial loads and detection of species diversity^55^. However, Virtanen *et al*. found significant differences in protein yield between patients regardless of the sampling device used^54^. Several recent studies of the vaginal microbiome used cervical-vaginal lavage (CVL) samples to identify microbial proteins by mass spectrometry^41,43,44^. The number of microbial proteins identified in these CVL studies was higher than the number we identified in the cell-free Pap test supernatant, ranging from 40 *Lactobacillus* proteins from 50 samples^41^, to 3334 bacterial proteins (188 species) from 688 samples^43^, and 689 bacterial proteins (64 species) from 41 samples^44^. This may be due to multiple factors, including the use of cell-free supernatants rather than CVL samples (which would include microbial cells) or the number of species/proteins represented in our custom database. Nevertheless, our studies demonstrate the utility of using Pap samples and cytology biobanks for studies of vaginal microbiota.

Because we used only the cell-free supernatant (and not the cellular component) of the Pap tests, only proteins that have already been released from bacterial cells, either because of secretion or lysis, would be detected, while the majority of bacterial cells (and proteins) may be present in the cell pellet. It is possible that protein extraction protocols that include the cell pellet would increase the yield of microbial peptides. However, an advantage to using just the cell-free residual Pap test fluid and omitting the cell pellet from the extraction step, is that it might be possible to identify the proteins that are secreted by the bacterial cells. In particular, the use of the fluid fixative allows for the potential to elucidate proteins important in cell-host interactions and bacterial response to environmental changes^19,51^.

Many of the proteins secreted by bacteria are enzymes used to acquire nutrients from the environment^19^. For example, the sn-glycerol-3-phosphate transport system permease protein, UgpE (found in NPap933) allows the direct uptake of glycerol-3-phosphate which can then enter the glycolysis pathway or be used for phospholipid synthesis^39^. We also identified several ATP-binding cassette (ABC) transporter complex proteins involved in the uptake of carbohydrates, as well as enzymes involved in carbohydrate metabolism. Indeed, in their recent analysis of vaginal microbiota and efficacy of the HIV microbicide tenofovir, Klatt *et al*.^43^ found that metabolic activity of *G*. *vaginalis* and other anaerobic species could deplete tenofovir levels faster than *Lactobacillus* dominant communities. In our study, we found multiple peptides from two haloacid dehalogenase (HAD) proteins, both from non-*Lactobacillus* species. HAD family proteins perform a variety of functions, including detoxification^56^ that could potentially be involved in tenofovir breakdown.

Similarly, Zevin *et al*.^44^ found a human proteomic signature from *G*. *vaginalis* dominated communities that demonstrated disrupted epithelial integrity, even in the absence of a clinical diagnosis of bacterial vaginosis. In addition, they showed that an unknown secreted factor was present in *G*. *vaginalis* culture supernatant and inhibited wound healing in an *in vitro* assay, but was not present in supernatant derived from *L*. *iners*^44^. In our study, we identified 10 of the 11 proteins that Zevin *et al*.^44^ determined were significantly differently expressed between *G*. *vaginalis* and *L*. *iners*, including the Elongation factor Tu and Glucose-6-phosphate isomerase proteins that were increased in the *G*. *vaginalis* dominated samples^44^. The HAD family hydrolase was the only *G*. *vaginalis* protein we identified with multiple peptides; however two *Acinetobacter baumanii* proteins that could affect the host epithelial cells were identified in the same samples. Acyl-CoA dehydrogenase is involved in butyrate synthesis^39^; γ-hydroxybutyrate (GBH) has been identified as a biomarker for bacterial vaginosis^57^. The second *A*. *baumanii* protein is a sel1 repeat family protein. The Sel1 repeat protein, LpnE, is a *Legionella pneumophila* virulence protein that promotes host cell infection by interaction with epithelial cells^58^.

Several recent studies have examined the bacterial composition of the human vagina relative to physical symptoms of vaginal atrophy or dysbiosis and found correlations with the expression of human genes or proteins^38,41,42,44^. While many of these human proteins were identified in our previous study, our sample size is not large enough to correlate the expression of specific human proteins with the bacterial taxa identified^21^.

Metaproteomics provides complementary and unique information on both the composition and functional status of the microbiome^59^, and could be used to assess whether there are core functions that are conserved across the different CST types as suggested by Ravel *et al*.^3^. Previous 16 S rRNA analyses have also been used to associate the presence of specific bacterial taxa with vaginal pH, bacterial vaginosis, HIV and HPV infections, and cervical cancer^3,9,40,49,60,61^. Metaproteomic analyses in studies such as those could provide additional insight into the mechanisms underlying disease, and will be even more powerful as the sequences of additional microbial genomes, from both pathogenic and commensal organisms become available.

In conclusion, these studies demonstrate that metaproteomic analysis of the microbiome can be effectively conducted with residual fixative from Pap test samples, even when the majority of the proteins are of human origin. Because the Pap test is routinely collected for cervical cancer detection, these samples have potential as a vast resource for understanding the vaginal microbiome and how it contributes to women’s health and disease.

## Methods

### Sample collection and mass spectrometry analysis

De-identified residual (waste) Pap test samples in SurePath^TM^ vials were obtained from the University of Minnesota BioNet Tissue Procurement Facility following approval by the University of Minnesota Institutional Review Board (Protocol 1101E94895) which does not require patient consent for use of de-identified clinical specimens. All experiments were performed in accordance with the relevant University of Minnesota Institutional Review Board guidelines and regulations. The collection of clinical specimens and processing for mass spectrometry has been previously described^21^. Briefly, cells collected from the ectocervix of healthy women undergoing routine screening for cervical cancer using the BD SurePath^TM^ liquid-based Pap test underwent automated processing, staining and examination by a pathologist for a clinical diagnosis. After one month, when the vials would have been discarded by the cytopathology laboratory, we were provided with vials from women at least 50 years old with normal cervical cytology and without obvious signs of infection or visible blood contamination. In all cases, the vials were stripped of identifying information and the remaining volume of SurePath^TM^ solution (approximately 2 mL) was used for analysis by mass spectrometry. The ages of the individuals were as follows: NPap94, 52 yrs; NPap134, 67 yrs; NPap137, 61 yrs; NPap929, 60 yrs; and NPap933, 59 yrs. SurePath^TM^ vials were vortexed to resuspend proteins and to release cells/proteins from the cervical sampling device that remained in each vial. The residual fluid was centrifuged for 5 min at 800 × g to pellet the cells, and the supernatant was removed for analysis. Equal volumes of the cell-free SurePath^TM^ fixative from 40 normal Pap test samples (median age of 58 years; ranging from 50–76 years) were pooled prior to trypsin digestion. Acetone precipitated proteins from the pooled and individual samples (~50–100 µg protein) were prepared for mass spectrometry by Filter Aided Sample Preparation^62^. Peptides were 2D fractionated offline by high pH reverse phase chromatography into 32 fractions. Fractions were concatenated into 16, and approximately 1 µg of each of the concatenated fractions was submitted to the LTQ Orbitrap Velos spectrometer (Thermo Fisher Scientific, Inc., Waltham, MA) as described previously^21^.

### Database searching and protein identification

High accuracy MS/MS data was searched using a two-step method^30,31^ against a database composed of microbial and human protein sequences. The database of microbial species of the urogenital tract [331,242 sequences from 130 bacterial proteomes plus *Candida* (Supplementary Table S1)] was generated from Human Microbiome Project reference genomes^32,33^ using an in-house program, *MicPrDB* (https://github.com/somiafiuni/MicPrDB) and merged with a “target” version of the Human Uniprot database and contaminant sequences, and 3-frame translated cDNA protein sequences [2,674,981 sequences in the 3-frame translated cDNA (EnSEMBL) database plus the target version of the human Uniprot database with contaminant sequences (November 2014)]. We included the 3-frame translated cDNA database for the first-step database search to ensure that spectra originating from variant peptides were not incorrectly assigned to microbial peptides (in native or modified form). In our prior work, we demonstrated that this approach yielded more accurate identifications^63^. The Paragon search algorithm^64^ in ProteinPilot™ was used for searching the mass spectrometry data within the Galaxy for proteomics (Galaxy-P) platform using the workflow depicted in Fig. 1.

For the second step search, accession numbers associated with all microbial peptide identifications from the first search were merged with the human database plus contaminants to create a “target-decoy” database, by appending the reversed protein sequences to the forward sequences, which was used for the calculation of the false discovery rate (FDR). This refined target-decoy database of human plus bacterial proteins and translated 3-frame cDNA sequences was used to search high mass accuracy peak lists with ProteinPilot™. These searches generated a.group file and a Proteomics System Performance Evaluation Pipeline (PSPEP) FDR report^47^. The.group file was used to generate a Peptide Summary Report, which with the PSPEP FDR report was used to identify microbial peptide sequences. Of the total measured spectra, 161,673 peptides were assigned from the combined human and microbial database at 5% local FDR; and a subset of these peptide-spectral matches were identified as distinct microbial peptides (see Supplementary Materials). The remaining spectra matched to human peptides and are previously described^21^ in ref.^21^.

All peptide-spectral matches were retained to allow for spectral counting, and text-formatting tools within Galaxy-P were used to generate a FASTA list for BLASTP searching. The FASTA list was split into a list of short sequences (≤30 amino acids), and a list of long sequences (>30 amino acids) and searched against the NCBInr database, using BLASTP parameters as previously described^31,46^. For short peptide sequences, the following parameters were used - set expectation value cutoff: 200 000; scoring matrix: PAM30; gap costs: Existence 9, Extension 1; word size for wordfinder algorithm: 2; multiple hits window size: 15; threshold: minimum score to add a word to the BLAST lookup table: 16; and use composition-based statistics: 0 or F. Longer peptides were subjected to the following BLASTP parameters: set expectation value cutoff: 10; scoring matrix: BLOSUM62; gap costs: Existence 11, Extension 1; word size for wordfinder algorithm: 3; multiple hits windowsize: 40; threshold: minimum score to add a word to the BLAST lookup table: 11; and use composition-based statistics: 2,T or D.

The BLAST-P output files were merged and exported from Galaxy-P. The BLAST-P and the peptide FASTA files for each sample were imported into the metagenomic/metaproteomic analysis tool MEGAN6^34^. MEGAN6 uses the Lowest Common Ancestor algorithm (LCA) to assign reads to taxa. It allows functional analyses using several different ontological systems. The greatest number of hits was obtained with InterPro2GO for functional classification to Gene Ontology categories and IPR protein families, and those results are reported here. The MEGAN6 LCA parameters used were**:** minScore = 25.0, maxExpected = 10.0, top Percent = 10.0, min Support = 1, weighted LCA Percent = 80.0. The MEGAN6 mapping file used for taxonomic assignments was acc2interpro-June2016X.abin, and the MEGAN6 mapping file used for InterPro2GO assignments was acc2interpro-June2016X.abin. Note that changes in the LCA parameters or the dates of the mapping files are likely to lead to some changes in assignments. In that sense, the assignments cannot be considered to be 100% definitive.

### Data availability

The MS proteomics data in this paper have been deposited in the ProteomeXchange Consortium (http://www.proteomexchange.org/) via the PRIDE partner repository^65^ with the dataset identifier PXD009596.

## Electronic supplementary material

Supplementary Tables and Figures

Data sets 1-6
