# Supplementary material for: Evaluating the potential of residual Pap test fluid as a resource for the metaproteomic analysis of the cervical-vaginal microbiome
Source: Sci Rep. 2018 Jul 18;8:10868. doi: 10.1038/s41598-018-29092-4 (PMC6052116; doi:10.1038/s41598-018-29092-4)

# Supplementary Materials

## Evaluating the potential of residual Pap test fluid as a resource for the metaproteomic analysis of the cervical-vaginal microbiome

Somaieh Afiuni-Zadeh, Kristin L. M. Boylan, Pratik D. Jagtap, Timothy J. Griffin
Joel D. Rudney, Marnie L. Peterson, and Amy P.N. Skubitz

**Supplementary Tables S1-S6:**
Table S1. Microbial database composition
Table S2. Genus counts-PSM and peptide
Table S3. Species counts
Table S4. InterPro Protein families
Table S5. Gene Ontology Categories
Table S6. Phylotypes

**Supplementary Figures:** Peptide Fractionation for selected peptides

**Supplementary Table S1: Microbial database composition**

**Organisms**

Acinetobacter baumannii ATCC 19606 = CIP 70.34
Actinobaculum massiliae ACS-171-V-Col2
Actinomyces coleocanis DSM 15436
Actinomyces neuii BVS029A5
Actinomyces turicensis ACS-279-V-Col4
Actinomyces urogenitalis DSM 15434
Aerococcus urinae ACS-120-V-Col10a
Aerococcus viridans ATCC 11563
Anaerococcus hydrogenalis ACS-025-V-Sch4
Anaerococcus lactolyticus ATCC 51172
Anaerococcus prevotii ACS-065-V-Col13
Anaerococcus tetradius ATCC 35098
Atopobium vaginae DSM 15829
Atopobium vaginae PB189-T1-4
Bifidobacterium breve ACS-071-V-Sch8b
Bifidobacterium dentium ATCC 27679
Bifidobacterium dentium JCVIHMP022
Brevibacterium mcbrellneri ATCC 49030

**Microbial database, continued**

Chryseobacterium gleum ATCC 35910
Clostridiales genomosp. BVAB3 str. UPII9-5
Corynebacterium aurimucosum ATCC 700975
Corynebacterium genitalium ATCC 33030
Corynebacterium glucuronolyticum ATCC 51866
Corynebacterium glucuronolyticum ATCC 51867
Corynebacterium jeikeium ATCC 43734
Corynebacterium lipophiloflavum DSM 44291
Corynebacterium pseudogenitalium ATCC 33035
Corynebacterium striatum ATCC 6940
Dialister micraerophilus DSM 19965
Dialister microaerophilus UPII 345-E
Enterococcus durans FB129-CNAB-4
Enterococcus faecalis ATCC 29200
Enterococcus faecalis HH22
Enterococcus faecalis TX0312
Enterococcus faecalis TX0635
Enterococcus faecalis TX0855
Eremococcus coleocola ACS-139-V-Col8
Escherichia coli 83972
Finegoldia magna ACS-171-V-Col3
Finegoldia magna ATCC 53516
Finegoldia magna BVS033A4
Finegoldia magna SY403409CC001050417
Fusobacterium nucleatum subsp. nucleatum ATCC 23726
Gardnerella vaginalis 315-A
Gardnerella vaginalis 409-05
Gardnerella vaginalis ATCC 14019
Gardnerella vaginalis HMP9231
Haemophilus parainfluenzae HK262
Lactobacillus coleohominis 101-4-CHN
Lactobacillus crispatus 125-2-CHN
Lactobacillus crispatus 214-1
Lactobacillus crispatus CTV-05
Lactobacillus crispatus FB049-03
Lactobacillus crispatus FB077-07
Lactobacillus crispatus JV-V01
Lactobacillus crispatus MV-1A-US
Lactobacillus crispatus MV-3A-US
Lactobacillus crispatus SJ-3C-US
Lactobacillus delbrueckii subsp. bulgaricus PB2003/044-T3-4
Lactobacillus fermentum 28-3-CHN
Lactobacillus gasseri 202-4
Lactobacillus gasseri 224-1
Lactobacillus gasseri JV-V03

**Microbial database, continued**

Lactobacillus gasseri MV-22
Lactobacillus gasseri SJ-9E-US
Lactobacillus gasseri SV-16A-US
Lactobacillus iners ATCC 55195
Lactobacillus iners DSM 13335
Lactobacillus iners LactinV 01V1-a
Lactobacillus iners LactinV 03V1-b
Lactobacillus iners LactinV 09V1-c
Lactobacillus iners LactinV 11V1-d
Lactobacillus iners LEAF 2052A-d
Lactobacillus iners LEAF 2053A-b
Lactobacillus iners LEAF 2062A-h1
Lactobacillus iners LEAF 3008A-a
Lactobacillus iners SPIN 1401G
Lactobacillus iners SPIN 2503V10-D
Lactobacillus iners UPII 143-D
Lactobacillus iners UPII 60-B
Lactobacillus jensenii 1153
Lactobacillus jensenii 115-3-CHN
Lactobacillus jensenii 269-3
Lactobacillus jensenii 27-2-CHN
Lactobacillus jensenii JV-V16
Lactobacillus jensenii SJ-7A-US
Lactobacillus johnsonii ATCC 33200
Lactobacillus oris PB013-T2-3
Lactobacillus salivarius ACS-116-V-Col5a
Lactobacillus vaginalis ATCC 49540
Megasphaera genomosp. type_1 str. 28L
Megasphaera sp. UPII 199-6
Mobiluncus curtisii ATCC 43063
Mobiluncus curtisii ATCC 51333
Mobiluncus curtisii subsp. curtisii ATCC 35241
Mobiluncus curtisii subsp. holmesii ATCC 35242
Mobiluncus mulieris 28-1
Mobiluncus mulieris ATCC 35239
Mobiluncus mulieris ATCC 35243
Mobiluncus mulieris FB024-16
Mycobacterium parascrofulaceum ATCC BAA-614
Peptoniphilus duerdenii ATCC BAA-1640
Peptoniphilus harei ACS-146-V-Sch2b
Peptoniphilus lacrimalis 315-B
Porphyromonas asaccharolytica PR426713P-I
Porphyromonas uenonis 60-3
Prevotella amnii CRIS 21A-A
Prevotella bivia JCVIHMP010

**Microbial database, continued**

Prevotella buccalis ATCC 35310
Prevotella denticola CRIS 18C-A
Prevotella disiens FB035-09AN
Prevotella oralis ATCC 33269
Prevotella timonensis CRIS 5C-B1
Propionibacterium sp. 409-HC1
Propionibacterium sp. 434-HC2
Proteus mirabilis ATCC 29906
Roseomonas cervicalis ATCC 49957
Sphingobacterium spiritivorum ATCC 33300
Sphingobacterium spiritivorum ATCC 33861
Staphylococcus aureus subsp. aureus MN8
Staphylococcus epidermidis BVS058A4
Staphylococcus lugdunensis ACS-027-V-Sch2
Staphylococcus simulans ACS-120-V-Sch1
Streptococcus bovis ATCC 700338
Streptococcus pseudoporcinus SPIN 20026
Streptococcus urinalis FB127-CNA-2
Treponema phagedenis F0421
Veillonella atypica ACS-049-V-Sch6
Veillonella parvula ACS-068-V-Sch12
Veillonella ratti ACS-216-V-Col6b
Candida albicans 3153A

| Supplementary Table S2: MEGAN6 Genus level assignments based on Peptide Spectral Matches and peptides* | | | | | | | | | | | | |
|---|---|---|---|---|---|---|---|---|---|---|---|---|
| | Npap94 | | Npap134 | | Npap137 | | Npap929 | | Npap933 | | NPapPool | |
| Genus | PSM | Peptides | PSM | Peptides | PSM | Peptides | PSM | Peptides | PSM | Peptides | PSM | Peptides |
| **Acinetobacter** | **3** | **2** | **2** | **1** | **2** | **2** | **6** | **1** | **3** | **3** | **6** | **2** |
| Actinobaculum | 1 | 1 | | | 1 | 1 | 1 | 1 | | | | |
| **Actinomyces** | **3** | **2** | **1** | **1** | **3** | **1** | **8** | **3** | **1** | **3** | **5** | **1** |
| **Aerococcus** | | | **1** | **1** | | | | | | | **5** | **4** |
| **Anaerococcus** | **1** | **1** | **2** | **1** | | | | | **3** | **3** | **1** | **1** |
| Atopobium | | | | | | | | | 1 | 1 | | |
| **Bifidobacterium** | **2** | **2** | | | | | **2** | **1** | **2** | **1** | **1** | **1** |
| Candida | | | 1 | 1 | 1 | 1 | | | 1 | 1 | 1 | 1 |
| **Corynebacterium** | **1** | **1** | **1** | **1** | **1** | **1** | **8** | **4** | **7** | **5** | **1** | **1** |
| Dialister | | | | | 1 | 1 | | | | | | |
| **Enterococcus** | **2** | **2** | | | | | | | | | | |
| Escherichia | | | | | | | | | 1 | 1 | | |
| **Fusobacterium** | | | **1** | **1** | **4** | **2** | | | | | **1** | **1** |
| **Gardnerella** | **2** | **1** | **1** | **1** | | | **6** | **1** | **1** | **1** | **15** | **1** |
| **Lactobacillus** | **3** | **3** | **6** | **5** | **6** | **4** | **4** | **3** | **31** | **17** | **86** | **57** |
| Mageeibacillus | | | | | | | | | 1 | 1 | | |
| Megasphaera | 1 | 1 | | | | | | | | | | |
| **Mobiluncus** | | | **1** | **1** | | | **5** | **4** | **1** | **1** | | |
| **Mycobacterium** | | | | | **3** | **1** | **2** | **2** | **3** | **1** | **1** | **1** |
| **Peptoniphilus** | | | | | | | **2** | **2** | **1** | **1** | **2** | **2** |
| **Porphyromonas** | | | | | | | **1** | **1** | **2** | **2** | | |
| **Prevotella** | | | | | **2** | **2** | **1** | **1** | | | | |
| **Propionibacterium** | | | | | | | | | | | **4** | **1** |
| Proteus | | | | | | | | | 1 | 1 | | |
| Roseomonas | | | | | | | | | 1 | 1 | 1 | 1 |
| **Sphingobacterium** | | | | | | | **1** | **1** | | | **2** | **1** |
| **Staphylococcus** | | | | | | | **3** | **2** | | | **1** | **1** |
| **Streptococcus** | | | | | **2** | **1** | | | | | **1** | **1** |
| **Treponema** | | | | | | | **5** | **1** | **3** | **2** | | |
| **Veillonella** | | | **3** | **3** | **2** | **1** | | | **1** | **1** | **1** | **1** |
| Total | 19 | 16 | 20 | 17 | 28 | 18 | 55 | 28 | 353 | 200 | 135 | 88 |

*Genera from Figure 2 with 2 or more PSMs assigned to at least one sample are shown in bold.

**Supplementary Table S3: MEGAN6 Species level assignments based on peptide spectral matches***

| Species | Npap94 | Npap134 | Npap137 | Npap929 | Npap933 | NPapPool |
|---|---|---|---|---|---|---|
| Acinetobacter baumannii | | | | | 1 | |
| Actinobaculum massiliense | | | 1 | 1 | | |
| **Actinomyces coleocanis** | | | | | **2** | |
| **Actinomyces turicensis** | | | | **3** | **2** | |
| **Actinomyces urogenitalis** | **3** | **1** | **3** | **5** | **6** | **5** |
| **Aerococcus urinae** | | **1** | | | | **3** |
| Aerococcus viridans | | | | | | 1 |
| Anaerococcus hydrogenalis | | | | | | 1 |
| Anaerococcus lactolyticus | 1 | 1 | | | | |
| Anaerococcus prevotii | | 1 | | | 1 | |
| **Anaerococcus tetradius** | | | | | **2** | |
| Atopobium vaginae | | | | | 1 | |
| **Bifidobacterium dentium** | | | | **2** | **2** | |
| Candida albicans | | 1 | | | 1 | 1 |
| Corynebacterium genitalium | | | | | 1 | |
| **Corynebacterium glucuronolyticum** | | **1** | | **4** | **3** | |
| Dialister micraerophilus | | | 1 | | | |
| Enterococcus faecalis | 1 | | | | | |
| Escherichia coli | | | | | 1 | |
| Fusobacterium nucleatum | | | | | | 1 |
| **Gardnerella vaginalis** | **2** | **1** | | **6** | **1** | **15** |
| **Lactobacillus coleohominis** | | | | | | **5** |
| **Lactobacillus crispatus** | **3** | | | | **35** | **16** |
| Lactobacillus equicursoris | | | | | 1 | |
| Lactobacillus fermentum | | | | 1 | | |
| **Lactobacillus iners** | | **2** | **1** | | **3** | **14** |
| **Lactobacillus jensenii** | | **2** | **3** | | | **1** |
| Lactobacillus psittaci | | | | | 1 | |
| **Lactobacillus salivarius** | | **1** | | | **3** | **1** |
| Lactobacillus vaginalis | | 1 | | | | |
| Mageeibacillus indolicus | | | | | 1 | |
| **Mobiluncus curtisii** | | **1** | | **3** | | |
| **Mobiluncus mulieris** | | | | **2** | **1** | |
| Mycobacterium parascrofulaceum | | | | 1 | | |
| Peptoniphilus lacrimalis | | | | | 1 | |
| Porphyromonas gingivalis | | | | | 1 | |
| Porphyromonas uenonis | | | | 1 | 1 | |
| Prevotella oralis | | | 1 | 1 | | |
| Roseomonas cervicalis | | | | | 1 | 1 |
| **Sphingobacterium spiritivorum** | | | | **1** | | **2** |
| **Staphylococcus epidermidis** | | | | **2** | | |
| Staphylococcus simulans | | | | 1 | | |
| **Treponema phagedenis** | | | | **5** | **3** | |
| Veillonella atypica | | 1 | | | 1 | |
| Total PSMs | 10 | 15 | 10 | 39 | 77 | 67 |

*Species from Figure 3 with 2 or more PSMs assigned to at least one sample are shown in bold.

**Supplementary Table S4: MEGAN6 InterPro Protein Families with 2 or more Peptide Spectral Matches***

| InterProt2GO | Protein Family | Glycolysis proteins |
|---|---|---|
| IPR013005 | 50S ribosomal protein uL4 | |
| IPR000120 | Amidase | |
| IPR002293 | Amino acid/polyamine transporter I | |
| IPR017959 | Aspartyl/glutamyl-tRNA(Asn/Gln) amidotransferase, subunit B /E | |
| **IPR010591** | **ATP11** | |
| IPR005294 | ATPase, F1 complex, alpha subunit | |
| IPR005722 | ATPase, F1 complex, beta subunit | |
| IPR022953 | ATP-dependent 6-phosphofructokinase | ✓ |
| **IPR010972** | **Beta-phosphoglucomutase** | ✓ |
| IPR018422 | Cation/H+ exchanger, CPA1 family** | |
| IPR000158 | Cell division protein FtsZ | |
| IPR017998 | Chaperone tailless complex polypeptide 1 (TCP-1) | |
| IPR002423 | Chaperonin Cpn60/TCP-1 family | |
| IPR004090 | Chemotaxis methyl-accepting receptor | |
| IPR003199 | Choloylglycine hydrolase/Peptidase C59 family** | |
| IPR011206 | Citrate lyase beta subunit-like | |
| IPR006472 | Citrate lyase, alpha subunit | |
| IPR004446 | D,D-heptose 1,7-bisphosphate phosphatase** | |
| **IPR001977** | **Dephospho-CoA kinase** | |
| IPR005743 | DNA gyrase, subunit A | |
| IPR002099 | DNA mismatch repair protein family | |
| IPR020667 | DNA mismatch repair protein, MutL | |
| **IPR002177** | **DNA-binding protein Dps** | |
| **IPR000941** | **Enolase** | ✓ |
| IPR019127 | Exosortase EpsH-related | |
| IPR017541 | Exosortase family protein XrtG | |
| IPR022781 | Flagellar biosynthesis protein, FliO** | |
| **IPR011289** | **Fructose-1,6-bisphosphate aldolase, class 2** | ✓ |
| IPR002347 | Glucose/ribitol dehydrogenase** | |
| **IPR014362** | **Glutamate dehydrogenase** | |
| IPR012220 | Glutamate synthase, eukaryotic** | |
| **IPR006095** | **Glutamate/phenylalanine/leucine/valine dehydrogenase** | |
| IPR004809 | Glutamine synthetase type I | |
| **IPR020831** | **Glyceraldehyde/Erythrose phosphate dehydrogenase family** | ✓ |
| IPR015902 | Glycoside hydrolase, family 13 | |
| IPR027031 | Glycyl-tRNA synthetase/DNA polymerase subunit gamma-2 | |
| IPR022955 | GMP synthase** | |
| IPR006357 | HAD-superfamily hydrolase, subfamily IIA | |
| IPR013126 | Heat shock protein 70 family | |
| IPR029033 | Histidine phosphatase superfamily | |
| **IPR000771** | **Ketose-bisphosphate aldolase, class-II** | |
| **IPR004903** | **Lactobacillus surface layer protein** | |
| **IPR011304** | **L-lactate dehydrogenase** | ✓ |
| IPR001557 | L-lactate/malate dehydrogenase | |
| IPR022934 | Manganese-dependent inorganic pyrophosphatase, probable | |
| IPR006138 | NADH-ubiquinone oxidoreductase, 20 Kd subunit | |

| InterProt2GO | Protein Family | Glycolysis proteins |
|---|---|---|
| IPR000183 | Ornithine/DAP/Arg decarboxylase | |
| IPR005322 | Peptidase C69, dipeptidase A | |
| IPR002610 | Peptidase S54, rhomboid | |
| IPR001328 | Peptidyl-tRNA hydrolase** | |
| **IPR011862** | **Phosphate binding protein** | |
| IPR001272 | Phosphoenolpyruvate carboxykinase, ATP-utilising | |
| IPR006318 | Phosphoenolpyruvate-utilising enzyme | |
| **IPR001672** | **Phosphoglucose isomerase (PGI)** | ✓ |
| **IPR001576** | **Phosphoglycerate kinase** | ✓ |
| IPR024692 | Phosphotransferase system, enzyme I | |
| IPR004704 | Phosphotransferase system, mannose/fructose/sorbose family IID component | |
| IPR018455 | Phosphotransferase system, sorbose subfamily IIB component, subgroup | |
| IPR001757 | P-type ATPase | |
| IPR027256 | P-type ATPase, subfamily IB | |
| IPR000103 | Pyridine nucleotide-disulphide oxidoreductase, class-II | |
| IPR001697 | Pyruvate kinase | ✓ |
| **IPR014092** | **Pyruvate oxidase** | |
| IPR004476 | Ribonuclease II/ribonuclease R** | |
| IPR028364 | Ribosomal protein L1/ribosomal biogenesis protein | |
| IPR004498 | Ribosomal protein L11 methyltransferase** | |
| IPR000911 | Ribosomal protein L11/L12 | |
| IPR005745 | Ribosomal protein L14, bacterial-type | |
| IPR000218 | Ribosomal protein L14b/L23e | |
| IPR000456 | Ribosomal protein L17 | |
| IPR001857 | Ribosomal protein L19 | |
| IPR002171 | Ribosomal protein L2 | |
| IPR005880 | Ribosomal protein L2, bacterial/organellar-type | |
| IPR013025 | Ribosomal protein L25/L23 | |
| IPR001854 | Ribosomal protein L29 | |
| IPR000597 | Ribosomal protein L3 | |
| IPR019927 | Ribosomal protein L3, bacterial/organelle-type | |
| IPR002136 | Ribosomal protein L4/L1e | |
| IPR000702 | Ribosomal protein L6 | |
| IPR019906 | Ribosomal protein L6, bacterial-type | |
| **IPR000206** | **Ribosomal protein L7/L12** | |
| IPR000110 | Ribosomal protein S1 | |
| IPR001848 | Ribosomal protein S10 | |
| IPR001971 | Ribosomal protein S11 | |
| IPR001892 | Ribosomal protein S13 | |
| IPR019980 | Ribosomal protein S13, bacterial-type | |
| IPR001865 | Ribosomal protein S2 | |
| IPR005706 | Ribosomal protein S2, bacteria/mitochondria/plastid | |
| IPR022801 | Ribosomal protein S4/S9 | |
| IPR000851 | Ribosomal protein S5 | |
| IPR005712 | Ribosomal protein S5, bacterial-type | |
| IPR000235 | Ribosomal protein S5/S7 | |
| IPR000529 | Ribosomal protein S6 | |
| IPR020814 | Ribosomal protein S6, plastid/chloroplast | |

| InterProt2GO | Protein Family | Glycolysis proteins |
|---|---|---|
| IPR005717 | Ribosomal protein S7, bacterial/organellar-type | |
| IPR000630 | Ribosomal protein S8 | |
| IPR000754 | Ribosomal protein S9 | |
| IPR004396 | Ribosome-binding ATPase YchF/Obg-like ATPase 1** | |
| IPR030165 | sn-glycerol-3-phosphate transport system permease protein UgpE | |
| IPR006059 | Solute-binding family 1 | |
| IPR002155 | Thiolase | |
| **IPR001585** | **Transaldolase** | |
| IPR004732 | Transaldolase type 2 | |
| IPR004540 | Translation elongation factor EFG/EF2 | |
| IPR001816 | Translation elongation factor EFTs/EF1B | |
| **IPR004541** | **Translation elongation factor EFTu/EF1A, bacterial/organelle** | |
| IPR000178 | Translation initiation factor aIF-2, bacterial-like | |
| IPR005215 | Trigger factor | |
| IPR000652 | Triosephosphate isomerase | ✓ |
| IPR004520 | tRNA modification GTPase MnmE | |
| IPR003720 | tRNA sulfurtransferase ThiI | |
| IPR005593 | Xylulose 5-phosphate/Fructose 6-phosphate phosphoketolase | |
| **IPR006175** | **YjgF/YER057c/UK114 family** | |
| Total number of PSMs (936) | | 363 |
| Total number of Protein Families Identified (113) | | 10 |

*The top twenty protein families with the most Peptide Spectral Matches in the Npap pool are shown in bold text.
**The eleven protein families with 2 or more Peptide Spectral Matches in at least 2 individual samples.

**Supplementary Table S5: MEGAN6 Gene Ontology Categories with 2 or more Peptide Spectral Matches in at least one sample***

| **GO Category** | **Cellular component organization** |
|---|---|
| GO:0043190 | ATP-binding cassette (ABC) transporter complex |
| GO:0005618 | **cell wall** |
| GO:0005694 | chromosome |
| GO:0005737 | cytoplasm |
| GO:0016020 | membrane |
| GO:0005739 | **mitochondrion** |
| GO:0005840 | **ribosome** |
| | **Biological Process** |
| **GO:0046034** | **ATP metabolic process** |
| **GO:0009058** | **biosynthetic process** |
| **GO:0005975** | **carbohydrate metabolic process** |
| **GO:0009056** | **catabolic process** |
| **GO:0006520** | **cellular amino acid metabolic process** |
| **GO:0016043** | **cellular component organization** |
| GO:0006935 | chemotaxis |
| **GO:0051186** | **cofactor metabolic process** |
| **GO:0006259** | **DNA metabolic process** |
| **GO:0006091** | **generation of precursor metabolites and energy** |
| GO:0006811 | ion transport |
| **GO:0006807** | **nitrogen compound metabolic process** |
| GO:0071941 | nitrogen cycle metabolic process |
| **GO:0009117** | **nucleotide metabolic process** |
| **GO:0055114** | **oxidation-reduction process** |
| **GO:0016310** | **phosphorylation** |
| GO:0019538 | protein metabolic process |
| **GO:0006950** | **response to stress** |
| GO:0016070 | RNA metabolic process |
| GO:0007165 | signal transduction |
| **GO:0044281** | **small molecule metabolic process** |
| GO:0006790 | sulfur compound metabolic process |
| **GO:0006412** | **translation** |
| **GO:0006810** | **transport** |
| | **Function** |
| GO:0016887 | ATPase activity |
| GO:0048037 | cofactor binding |
| GO:0003924 | GTPase activity |
| GO:0016787 | **hydrolase activity** |
| GO:0016810 | hydrolase activity, acting on carbon-nitrogen (but not peptide) bonds |
| GO:0016788 | hydrolase activity, acting on ester bonds |
| GO:0016853 | **isomerase activity** |
| GO:0016301 | **kinase activity** |
| GO:0016874 | ligase activity |
| GO:0016829 | **lyase activity** |
| GO:0046872 | **metal ion binding** |
| GO:0004518 | nuclease activity |
| GO:0003676 | **nucleic acid binding activity** |
| GO:0000166 | nucleotide binding |
| GO:0016491 | **oxidoreductase activity** |
| GO:0008233 | peptidase activity |
| GO:0016791 | phosphatase activity |
| GO:0034062 | RNA polymerase activity |
| GO:0000988 | **transcription factor activity, protein binding** |
| GO:0016740 | **transferase activity** |
| GO:0016746 | transferase activity, transferring acyl groups |
| GO:0016757 | transferase activity, transferring glycosyl groups |
| GO:0005215 | transporter activity |

*Categories shown in bold type had two or more Peptide Spectral Matches in the Npap pool.

**Supplementary Table S6: Phylotypes with 2 or more Peptide Spectral Matches in at least one sample**

| | Npap94 | Npap134 | Npap137 | Npap929 | Npap933 | NPapPool |
|---|---|---|---|---|---|---|
| Acinetobacter | 3 | 2 | 2 | 6 | 3 | 6 |
| Actinomyces coleocanis | | | | | 2 | |
| Actinomyces turicensis | | | | 3 | 2 | |
| Actinomyces urogenitalis | 3 | 1 | 3 | 5 | 6 | 5 |
| Aerococcus | | | | | | 2 |
| Aerococcus urinae | | 1 | | | | 3 |
| Anaerococcus | | 2 | | | 1 | 1 |
| Anaerococcus tetradius | | | | | 2 | |
| Bifidobacterium | 2 | | | | | 1 |
| Bifidobacterium dentium | | | | 2 | 2 | |
| Corynebacterium | 1 | | 1 | 4 | 4 | 1 |
| Corynebacterium glucuronolyticum | | 1 | | 4 | 3 | |
| Enterococcus | 2 | | | | | |
| Fusobacterium | | 1 | 4 | | | 1 |
| Gardnerella vaginalis | 2 | 1 | | 6 | 1 | 15 |
| Lactobacillus | | 1 | 2 | 4 | 269 | 49 |
| Lactobacillus coleohominis | | | | | | 5 |
| Lactobacillus crispatus | 3 | | | | 35 | 16 |
| Lactobacillus iners | | 2 | 1 | | 3 | 14 |
| Lactobacillus jensenii | | 2 | 3 | | | 1 |
| Lactobacillus salivarius | | 1 | | | 3 | 1 |
| Mobiluncus curtisii | | 1 | | 3 | | |
| Mobiluncus mulieris | | | | 2 | 1 | |
| Mycobacterium | | | 3 | 2 | 3 | 1 |
| Peptoniphilus | | | | 2 | 1 | 2 |
| Porphyromonas | | | | 1 | 2 | |
| Prevotella | | | 2 | 1 | | |
| Propionibacterium | | | | | | 4 |
| Sphingobacterium spiritivorum | | | | 1 | | 2 |
| Staphylococcus | | | | 1 | | 1 |
| Staphylococcus epidermidis | | | | 2 | | |
| Streptococcus | | | 2 | | | 1 |
| Treponema phagedenis | | | | 5 | 3 | |
| Veillonella | | 3 | 2 | | 1 | 1 |
| Total PSMs | 16 | 19 | 25 | 54 | 347 | 133 |
| Total Phylotypes | 7 | 13 | 11 | 18 | 20 | 22 |

# NPap94

## AEDADDLSPSIVVSR

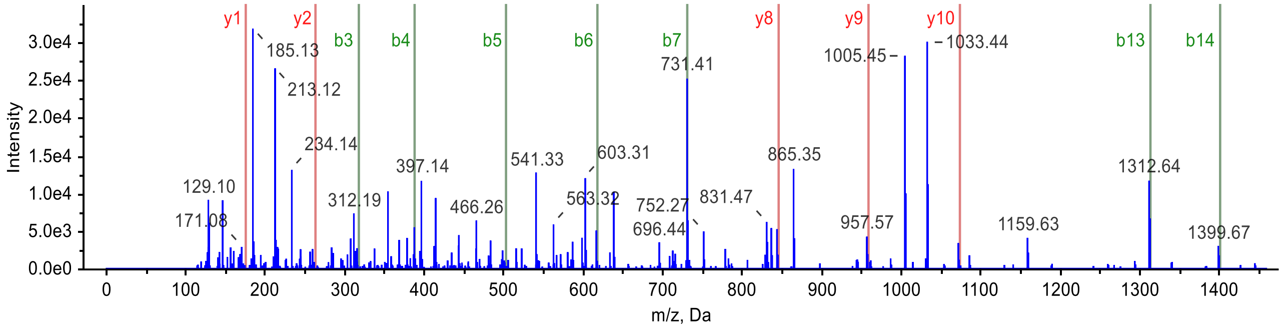


## QAAAAPTQPAPK

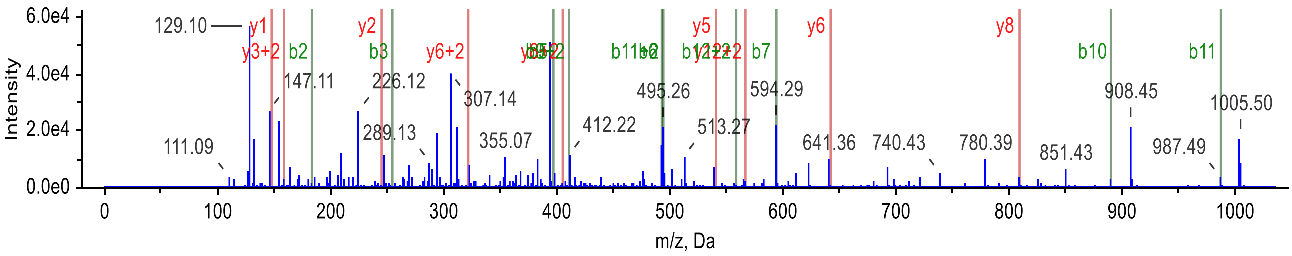


## SAGLNPVKSCK

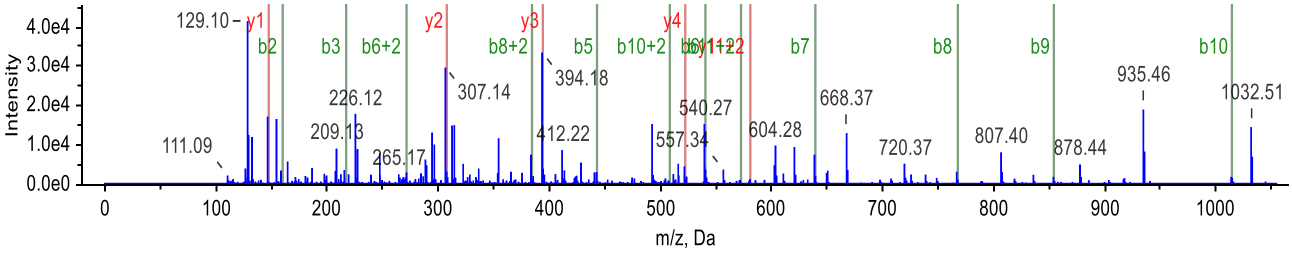


## SLMLGKEGEGLK

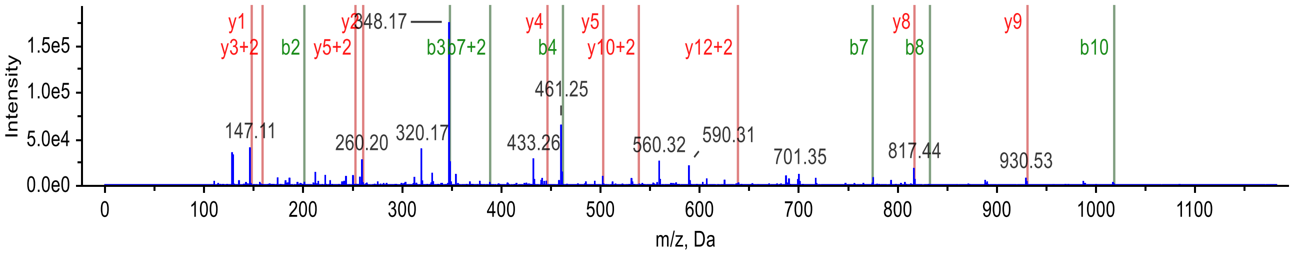

# NPap134

## AEDADDLSPSIVVSR

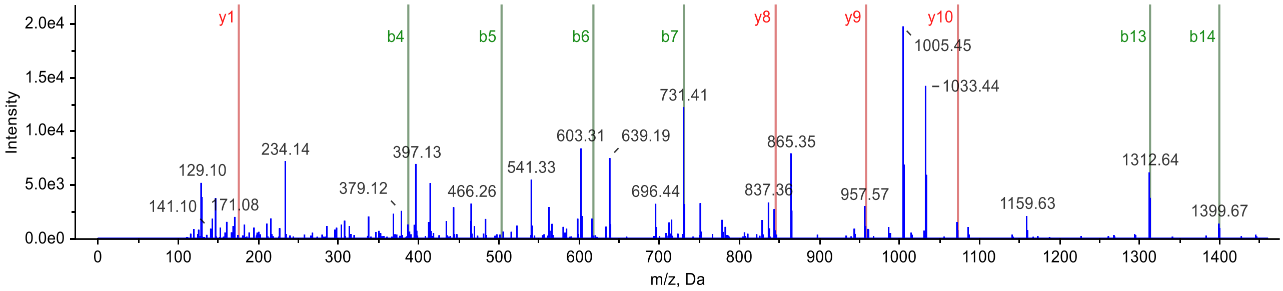


## DGIEPILEK

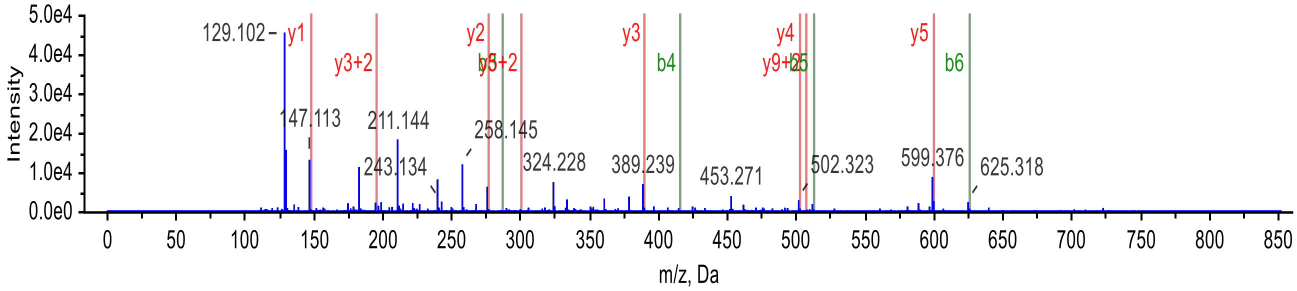


## IADLQWVDGAK

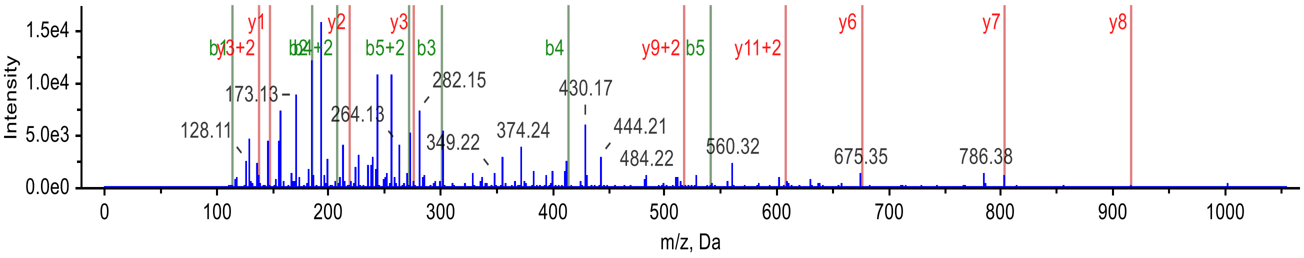


## LEEAGLTKNK

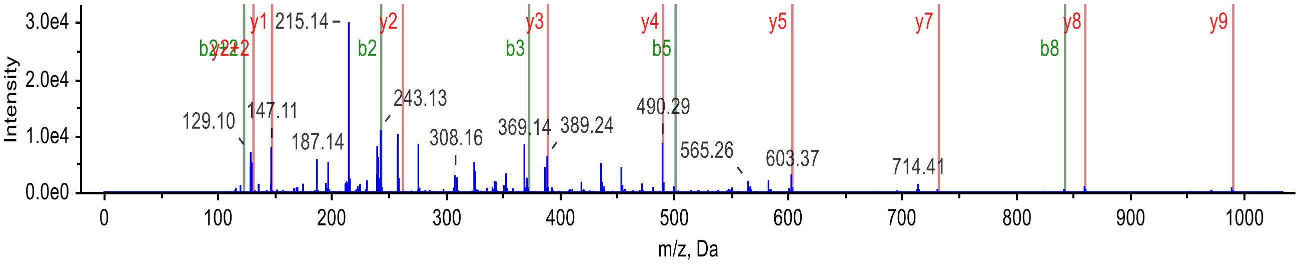

# NPap134, cont.

## QAAAAPTQPAPK

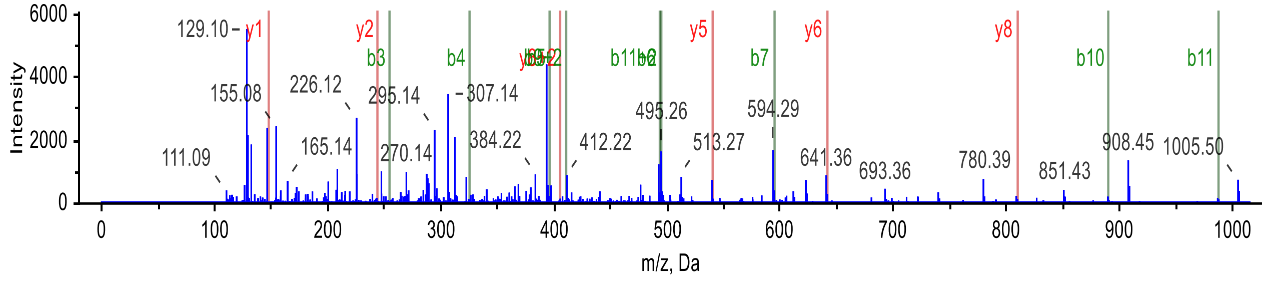


## QAEYDLITK

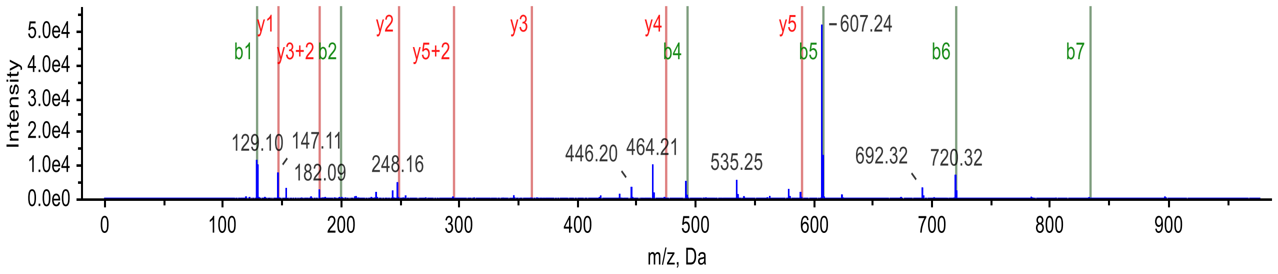


## SAGLNPVKSCK

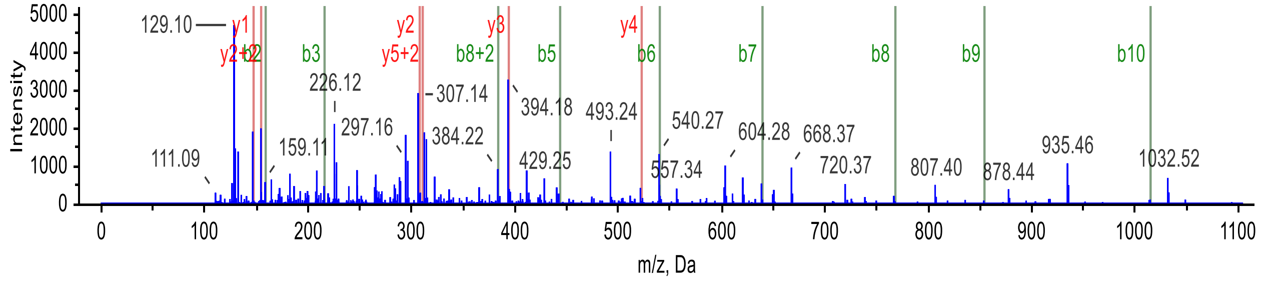

# NPap137

## AEDADDLSPSIVVSR

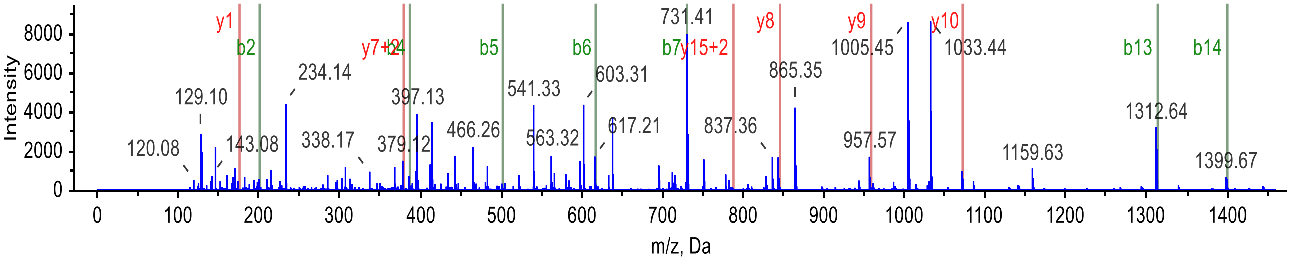


## LEEAGLTKNK

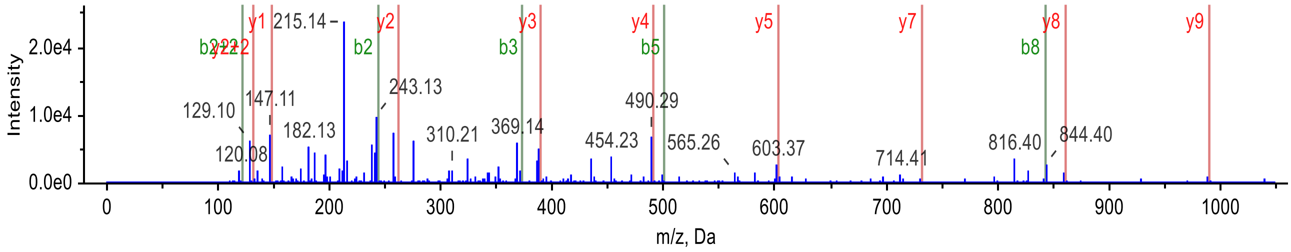


## IADLQWVDGAK

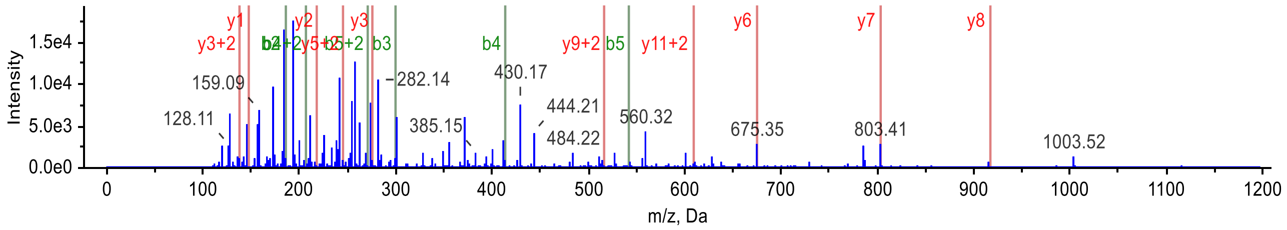


## QAAAAPTQPAPK

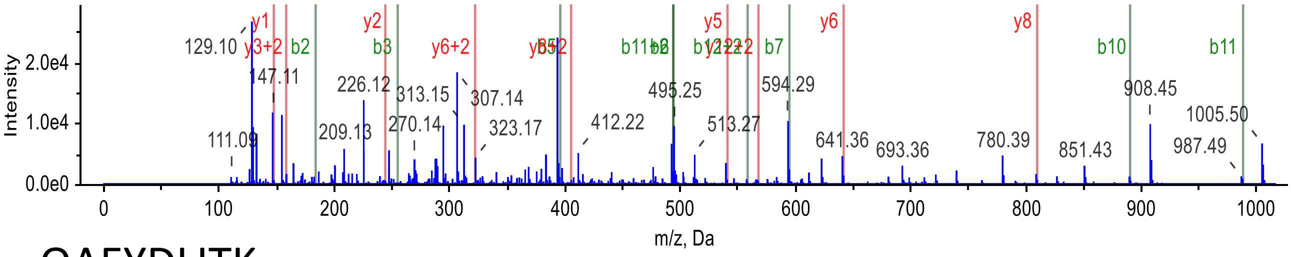


## QAEYDLITK

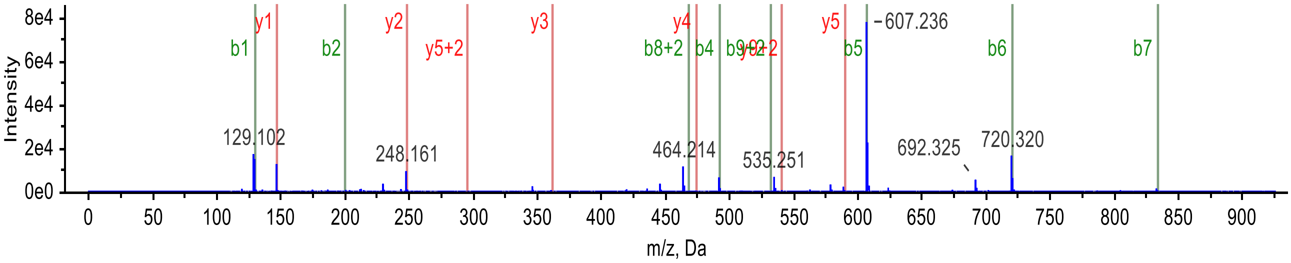

# NPap929

## AEDADDLSPSIVVSR

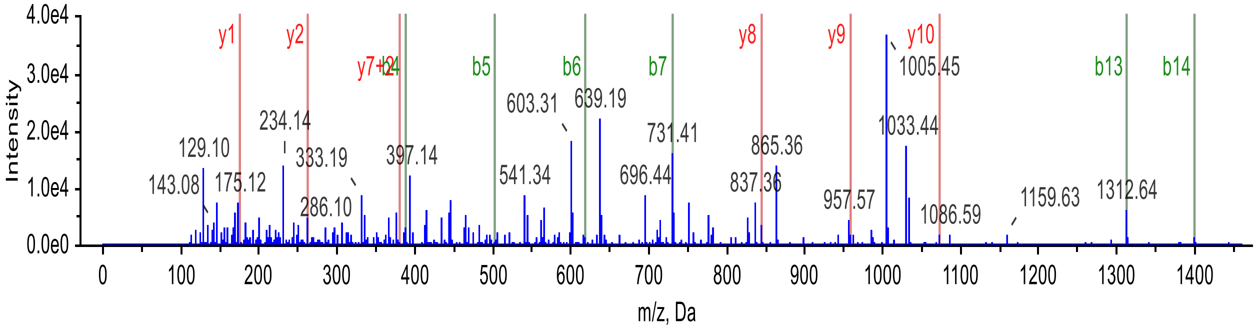


## DGIEPILEK

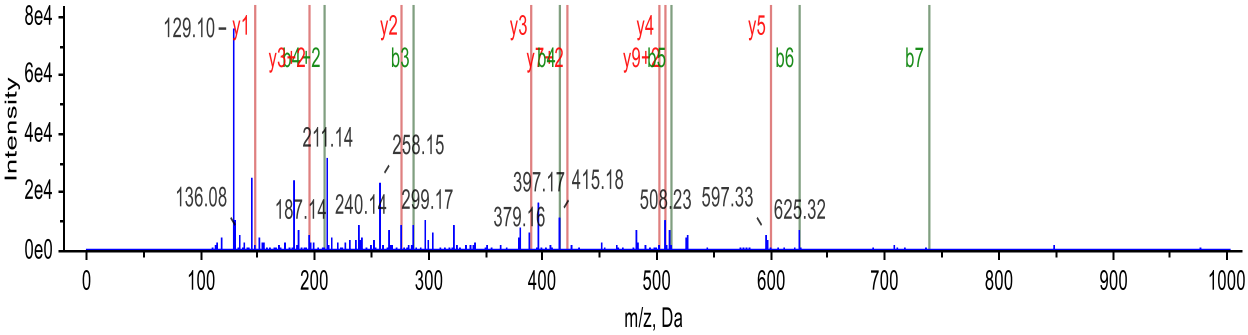


## EAAIAFSAIEK

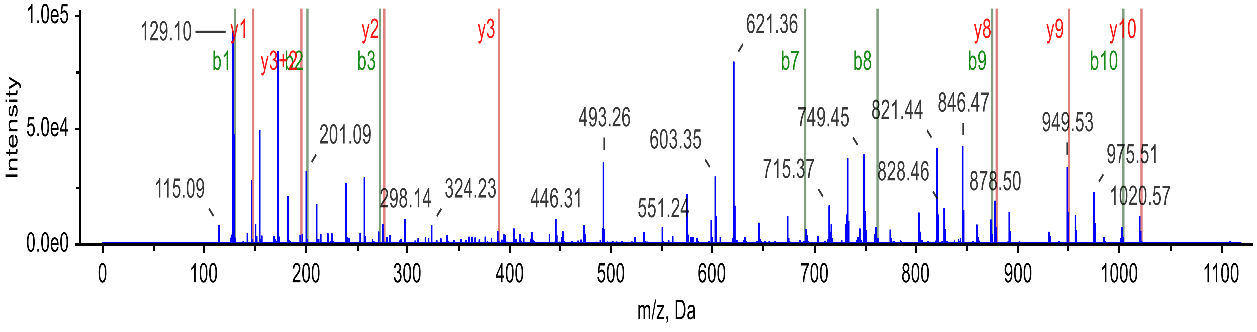

# NPap929, cont.

## ITWGEMEK

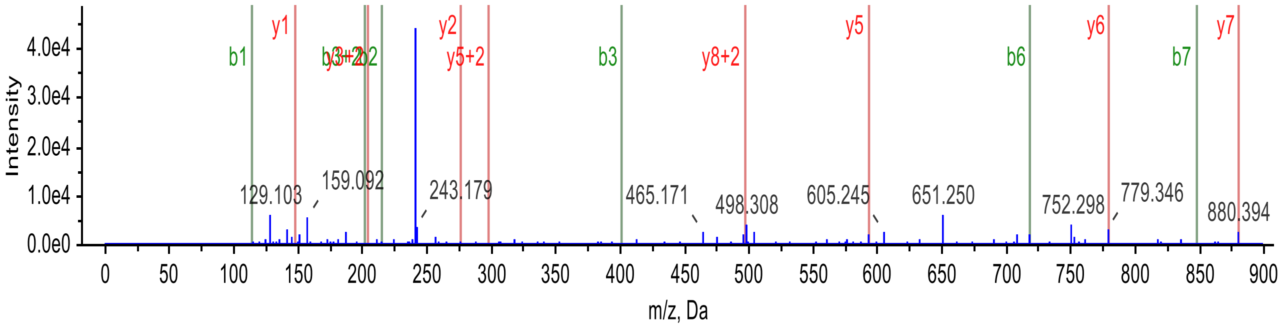


## MLLFAGGDLR

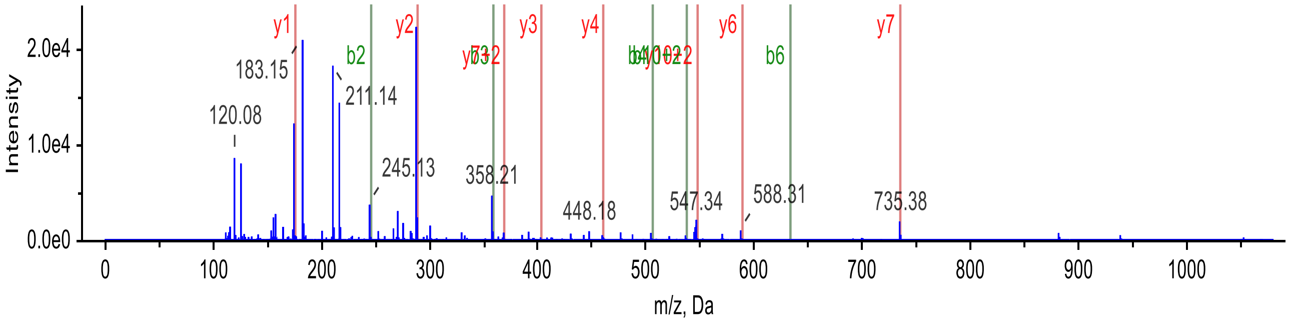


## SLMLGKEGEGLK

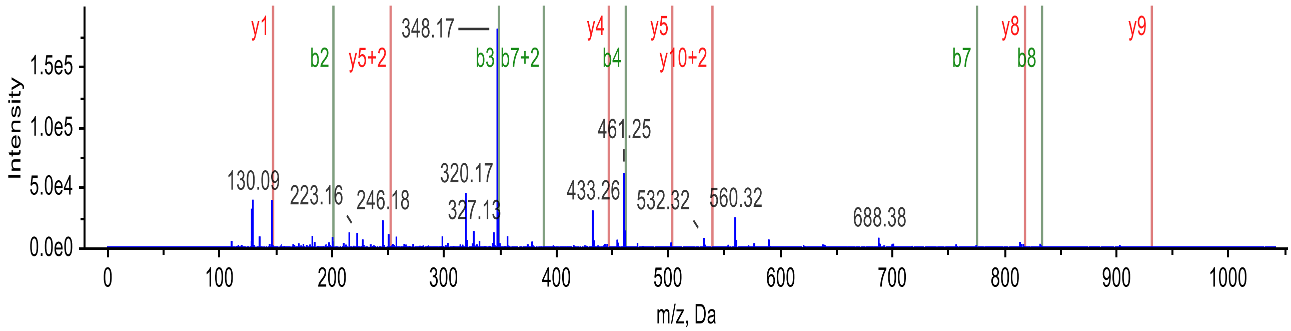

# NPap933

## AEDADDLSPSIVVSR

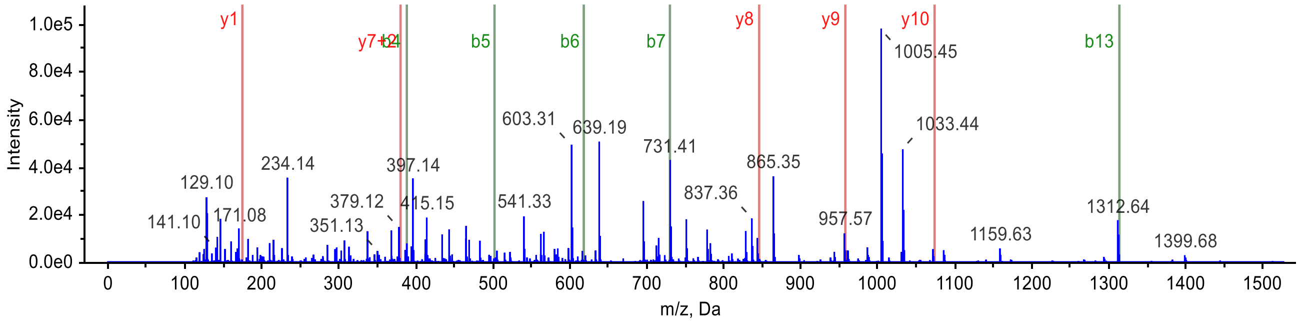


## AVVANSAEEANSK

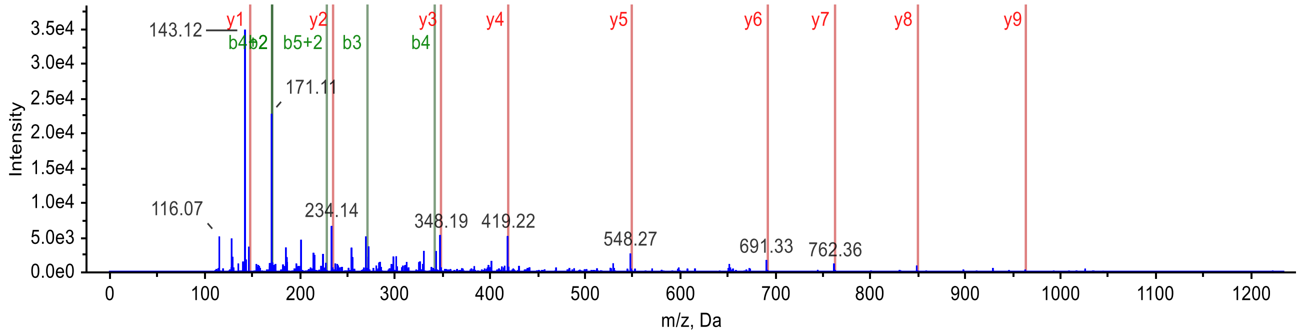


## DGDTVYVADQTR

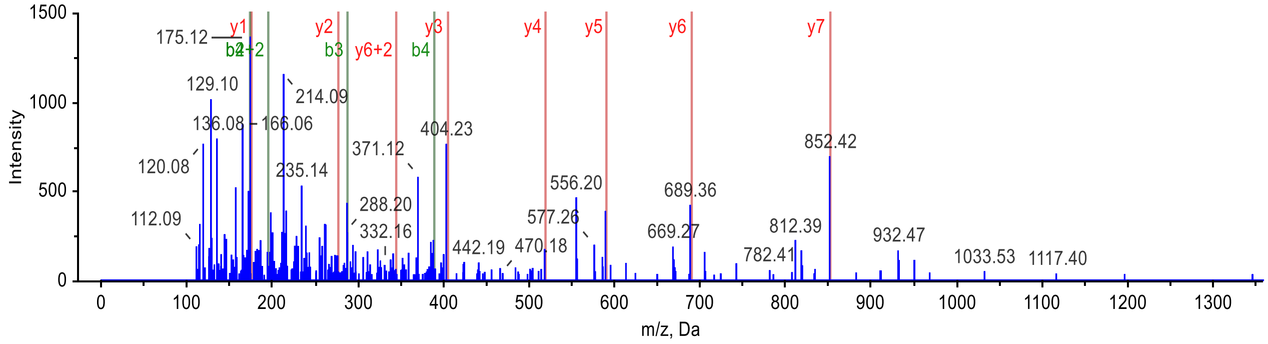


## DLYNEETAENVR

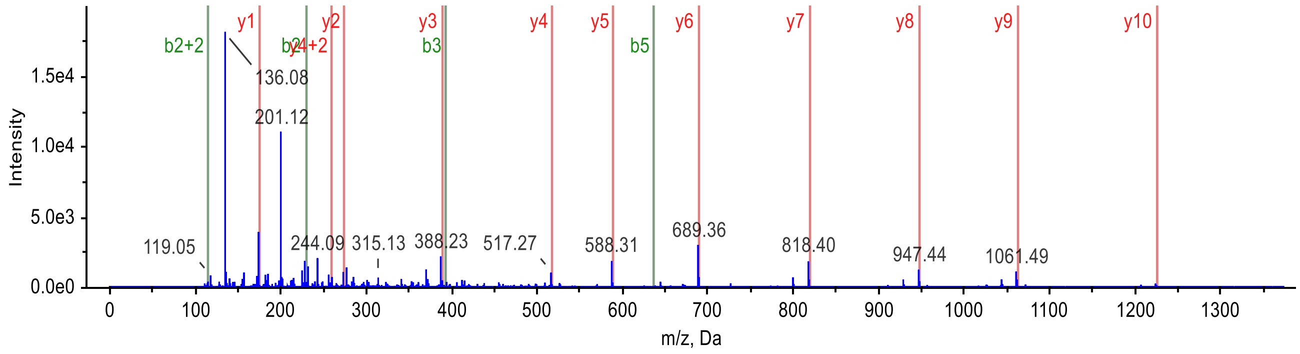

# NPap933, cont.

## DNVAATEFSNDASR

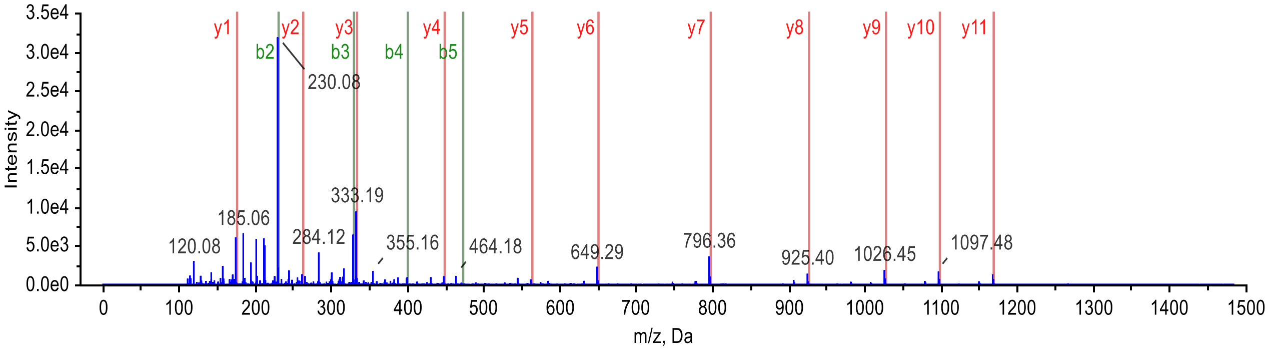


## EAAIAFSAIEK

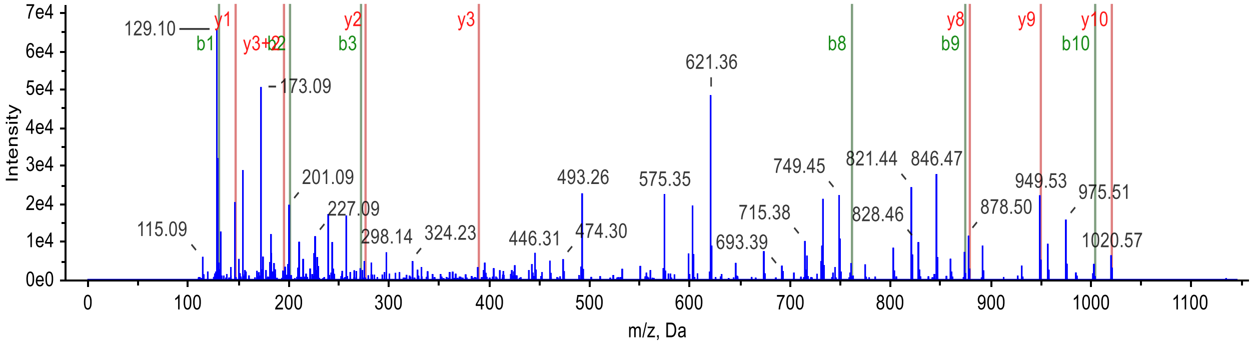


## EAAIAFSAIEK

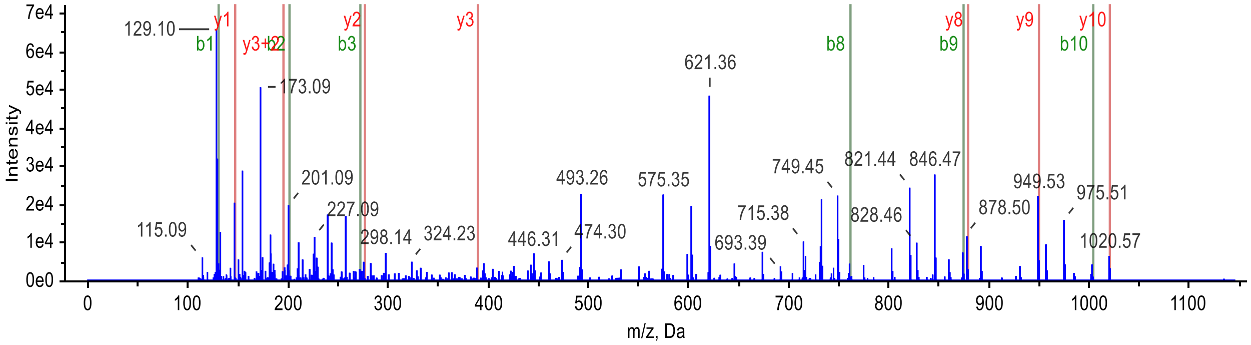


## ITWGEMEK

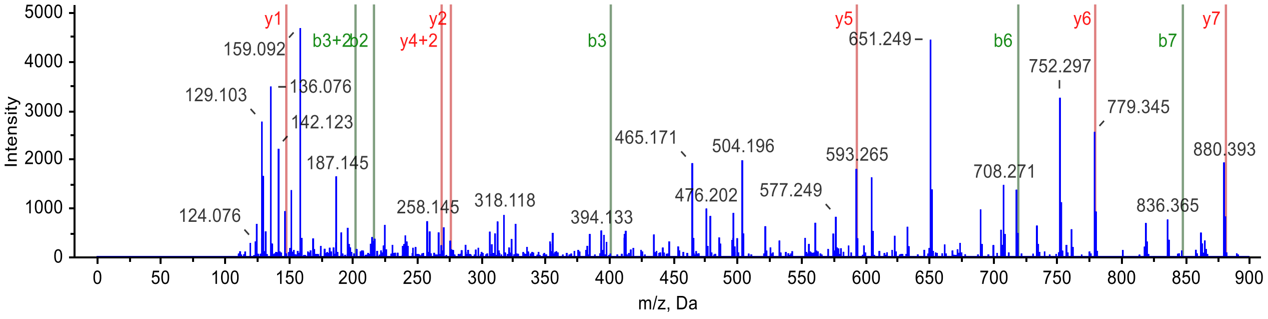

# NPap933, cont.

## LAFFPDDVDHD

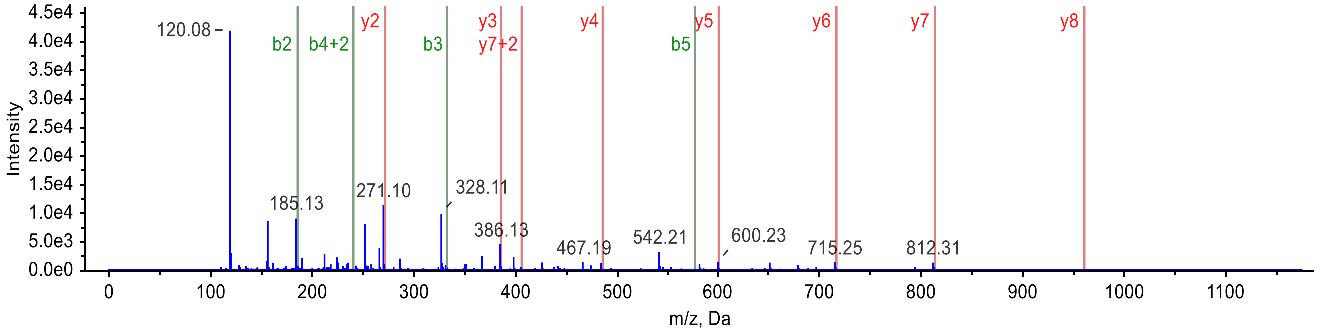


## LIVDDLDVK

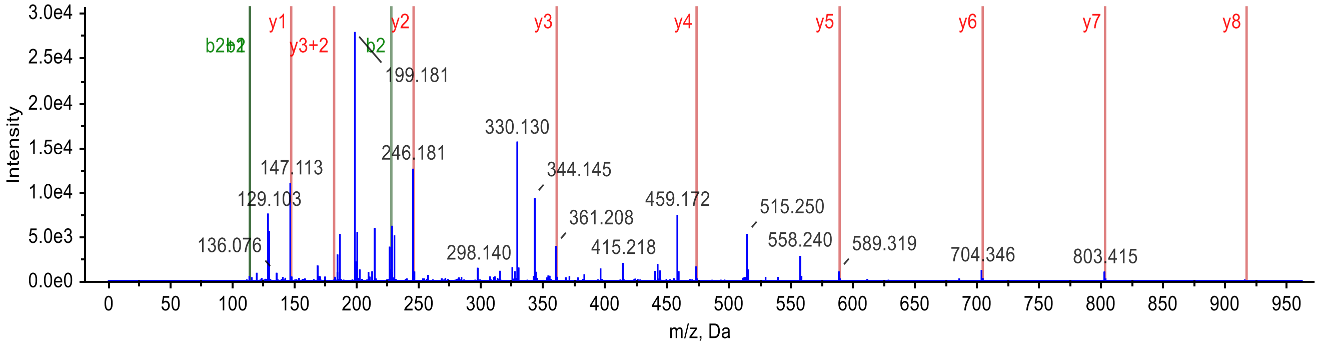


## MLLFAGGDLR

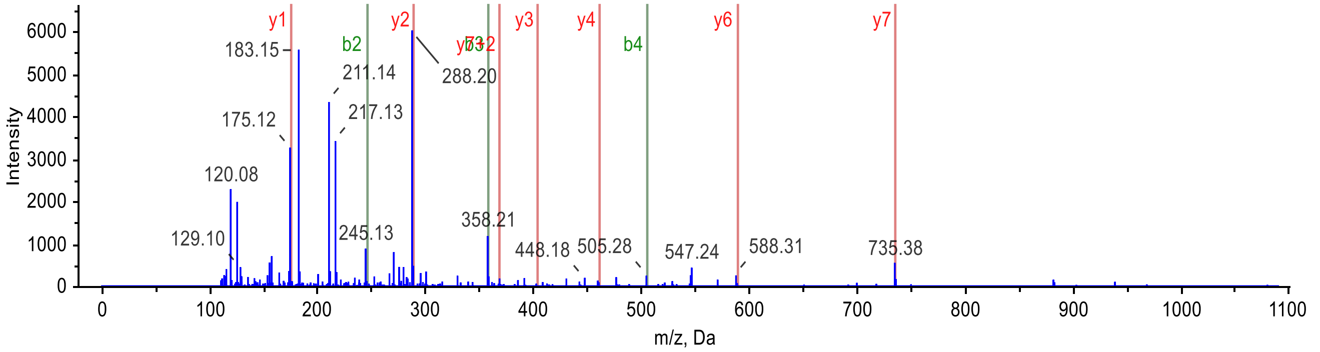


## SEFDVELTEAGQEK

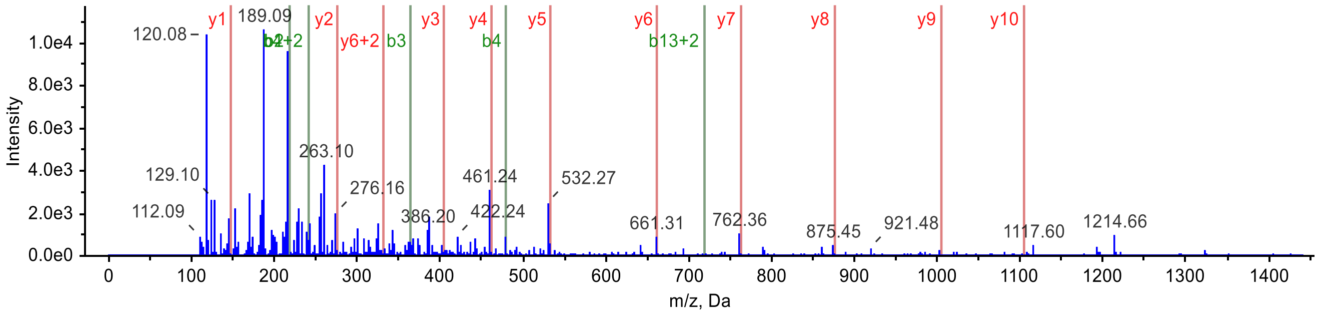

# NPap933, cont.

## TIHAYTGTQMTLDGPSR

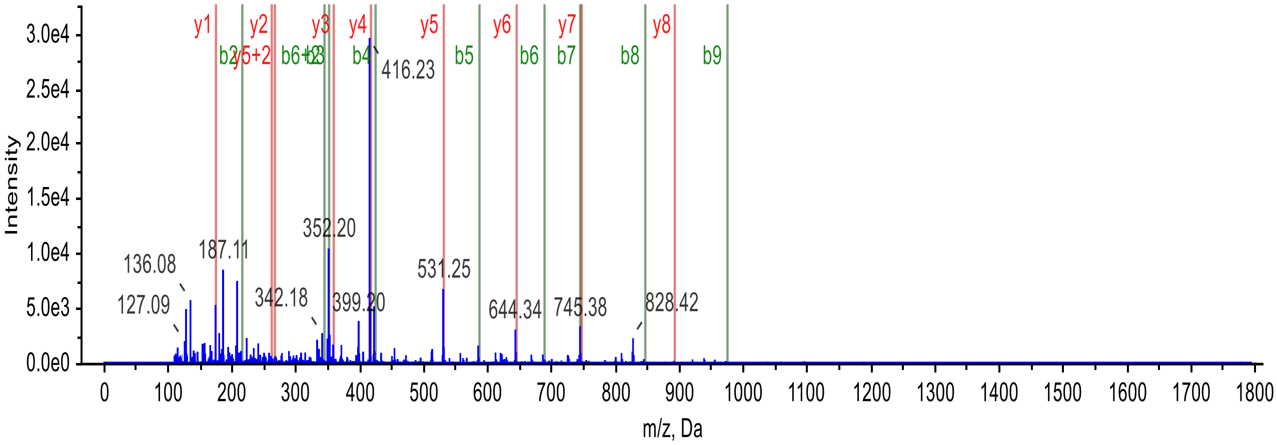


## VPVPDGSETELVSILSK

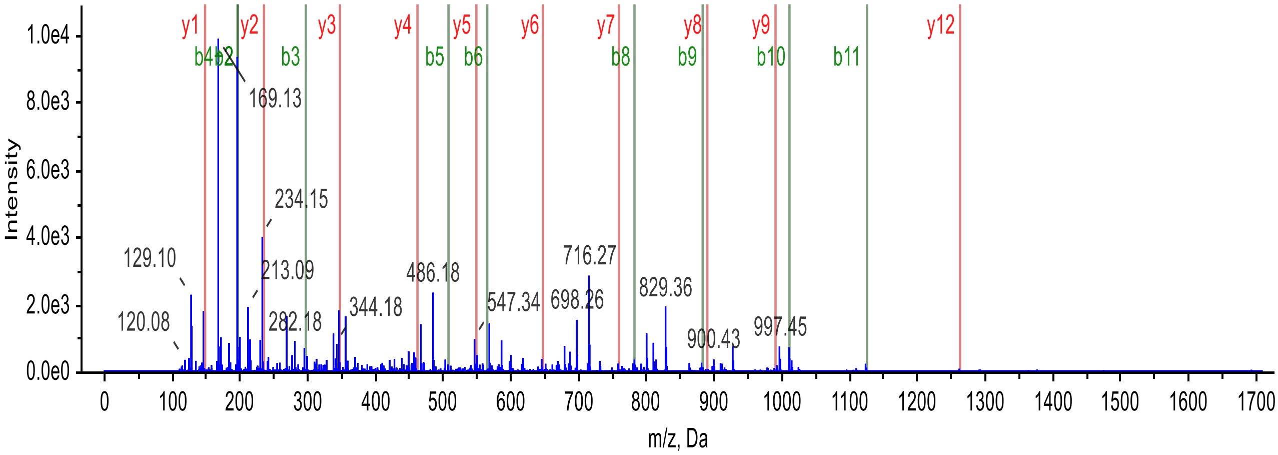


## YDTTHGTFNHEVSSTEDSIVVDGK

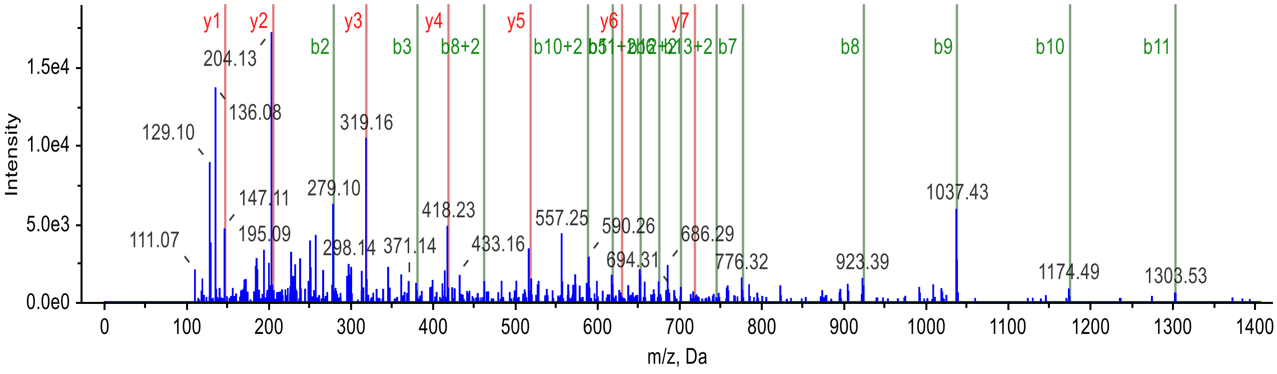

# NPap Pool

## AEDADDLSPSIVVSR

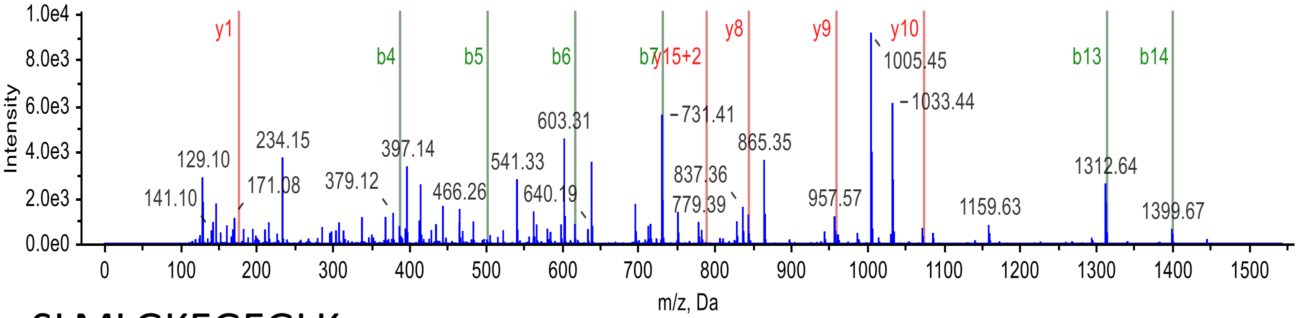


## SLMLGKEGEGLK

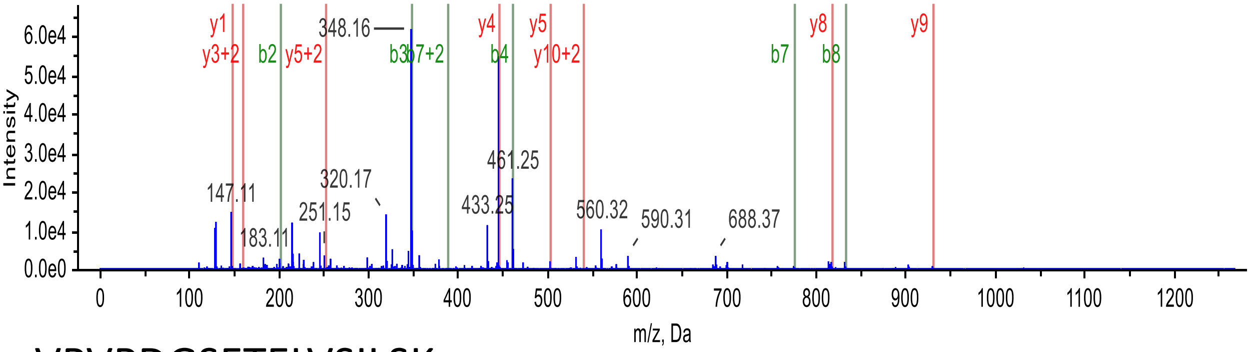


## VPVPDGSETELVSILSK

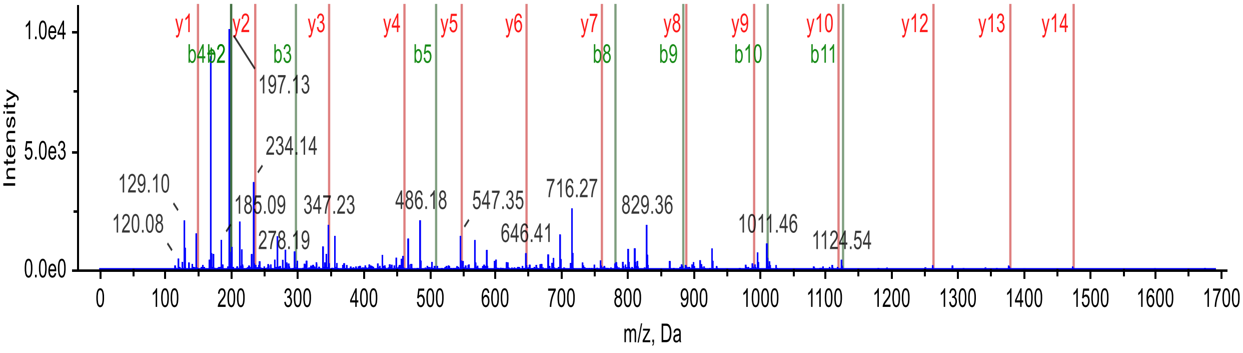


## YDTTHGTFNHEVSSTEDSIVVDGK

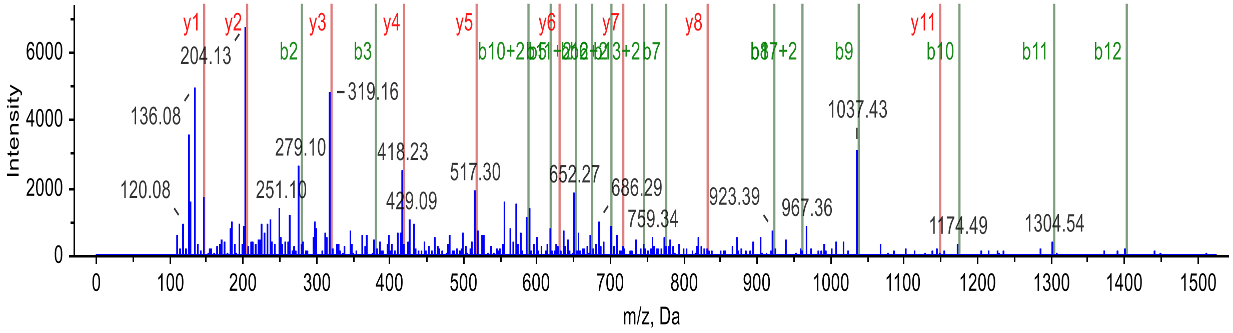

# NPap Pool

## AEDADDLSPSIVVSR

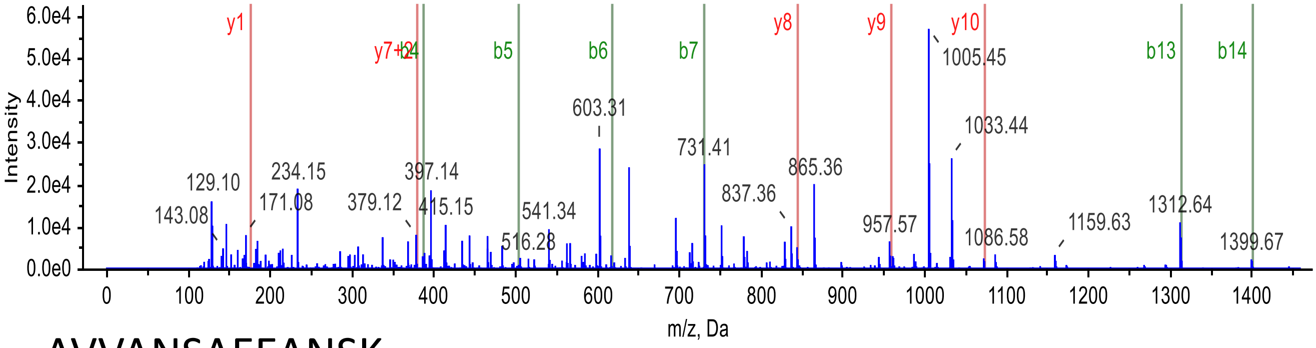


## AVVANSAEEANSK

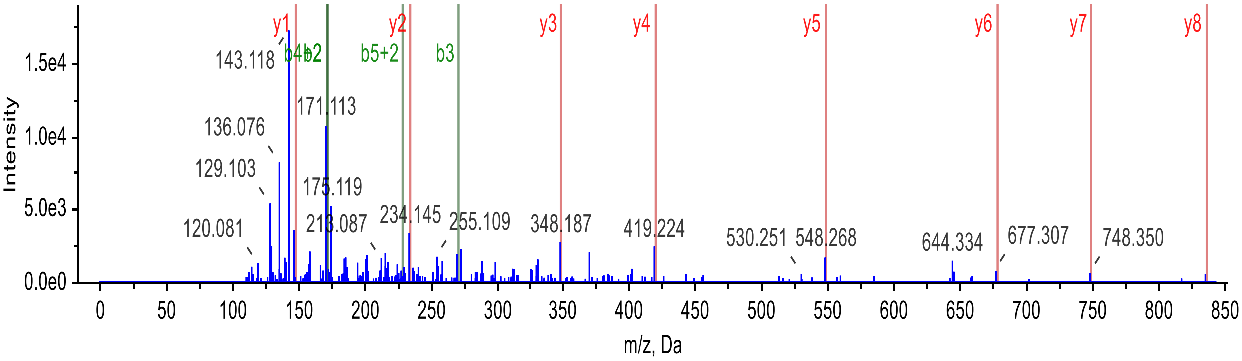


## DGDTVYVADQTR

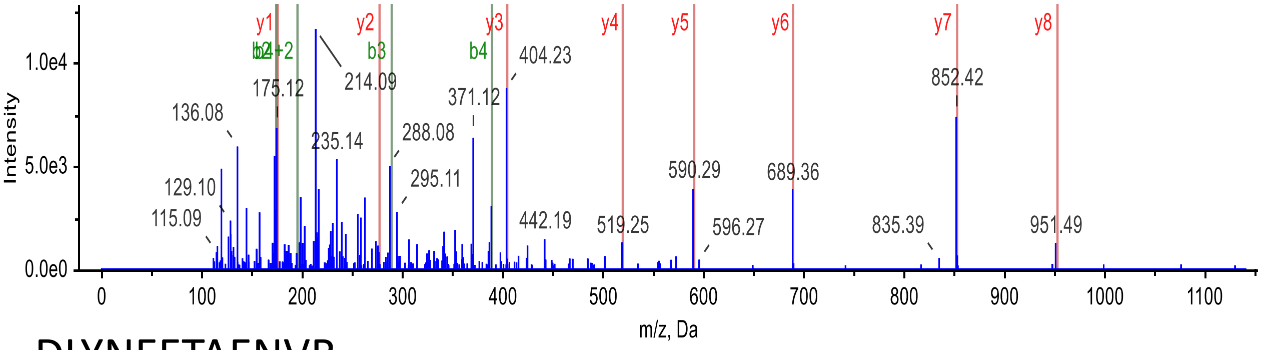


## DLYNEETAENVR

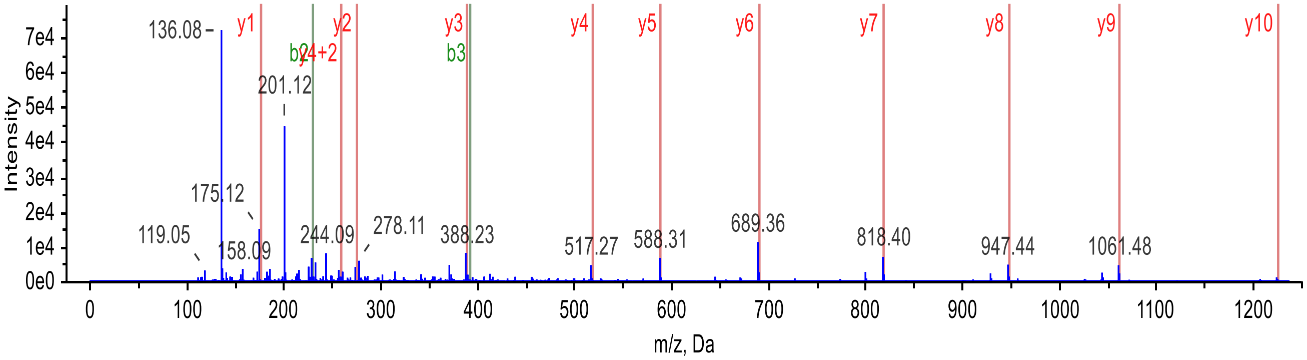

# NPap Pool, cont.

## EADYIVPTTAELK

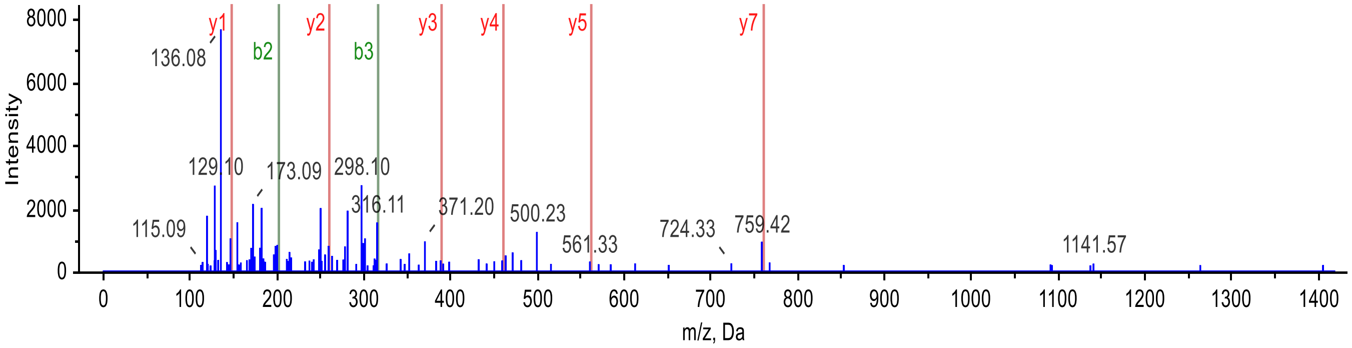


## LAFFPDDVDHD

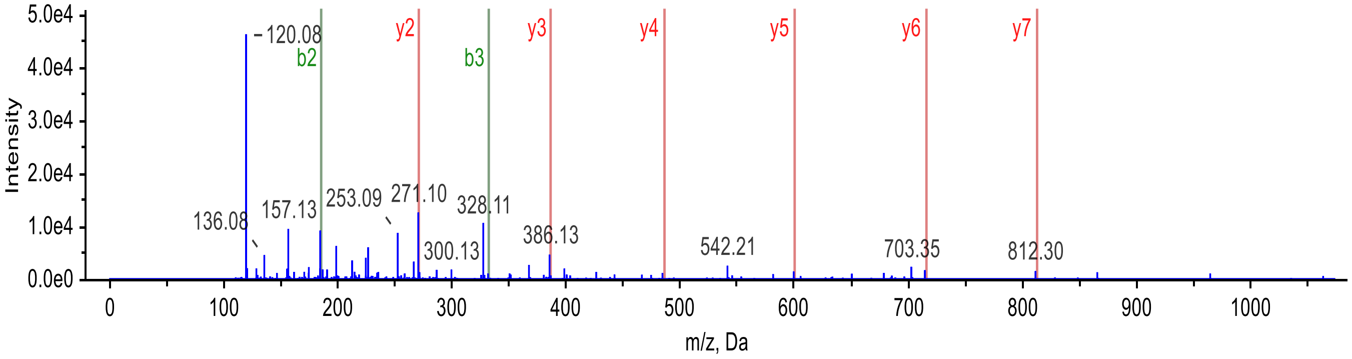


## LIVDDLDVK

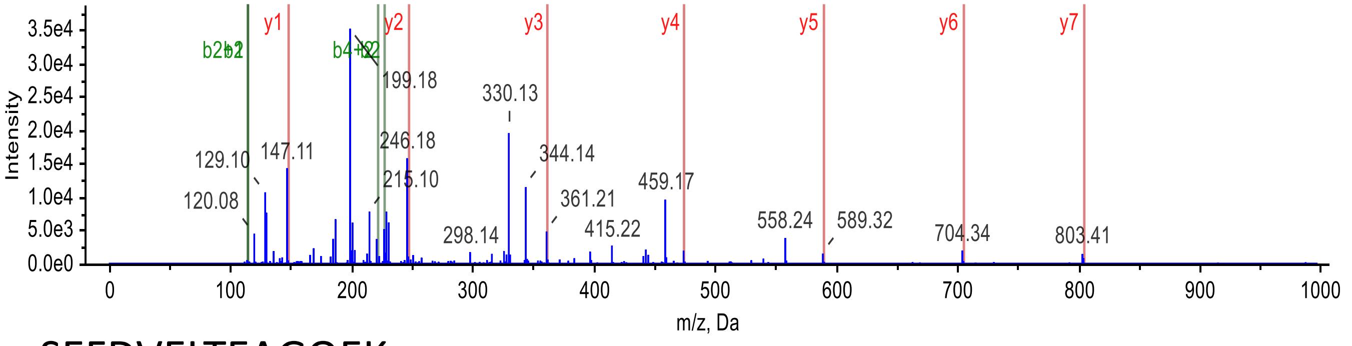


## SEFDVELTEAGQEK

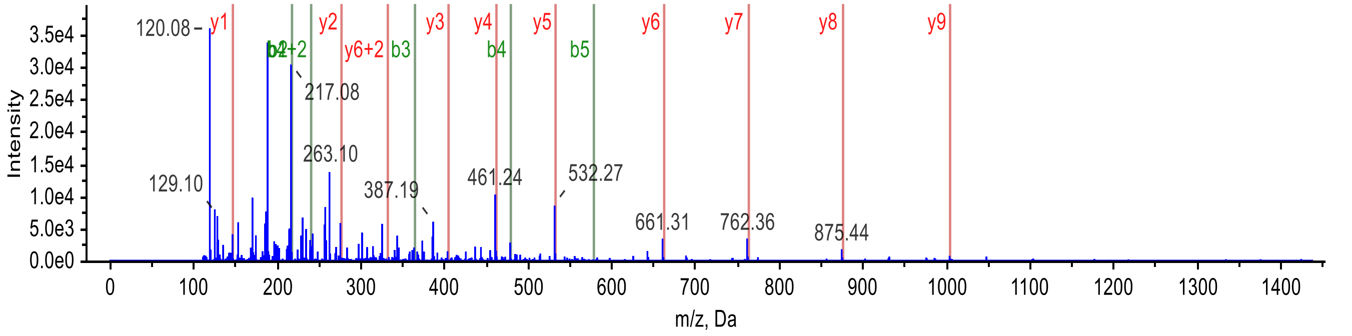

# NPap Pool, cont.

## SLMLGKEGEGLK

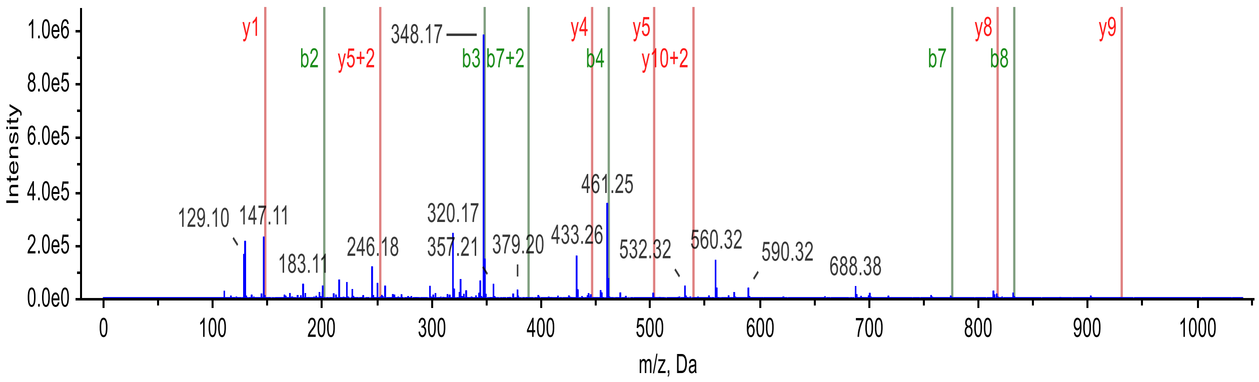


## TIHAYTGTQMTLDGPSR

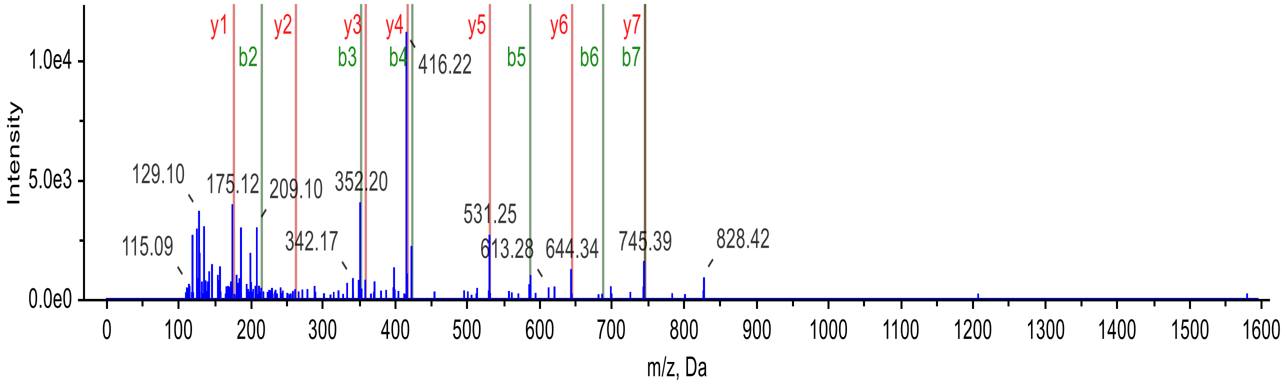


## YDTTHGTFNHEVSSTEDSIVVDGK

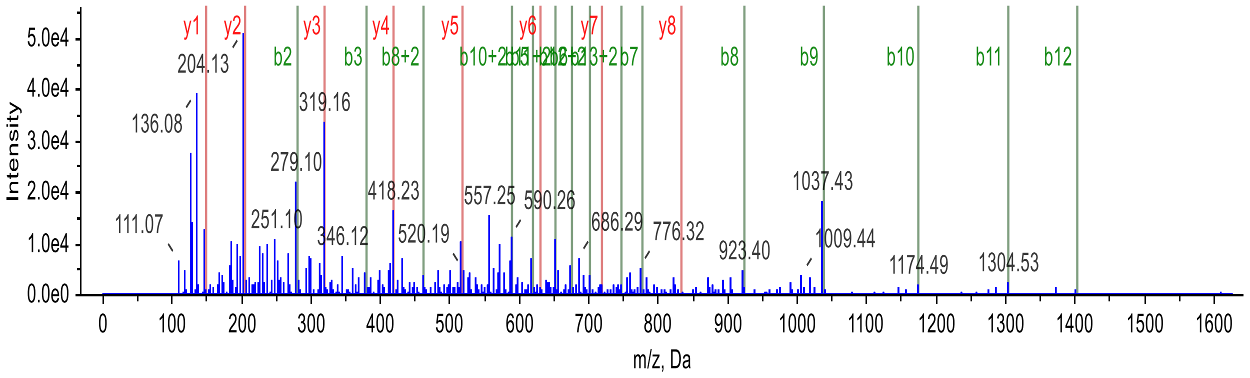

Supplement: Supplementary file 1 — Supplementary Tables and Figures [file 41598_2018_29092_MOESM1_ESM.pdf]
